# White matter microstructure and sleep-wake disturbances in individuals at ultra-high risk of psychosis

**DOI:** 10.3389/fnhum.2022.1029149

**Published:** 2022-10-28

**Authors:** Jesper Ø. Rasmussen, Dorte Nordholm, Louise B. Glenthøj, Marie A. Jensen, Anne H. Garde, Jayachandra M. Ragahava, Poul J. Jennum, Birte Y. Glenthøj, Merete Nordentoft, Lone Baandrup, Bjørn H. Ebdrup, Tina D. Kristensen

**Affiliations:** ^1^Centre for Neuropsychiatric Schizophrenia Research, Mental Health Centre Glostrup, Copenhagen University Hospital – Mental Health Services CPH, Copenhagen, Denmark; ^2^Copenhagen Research Centre for Mental Health, Mental Health Centre Copenhagen, Copenhagen University Hospital – Mental Health Services CPH, Copenhagen, Denmark; ^3^Department of Psychology, Faculty of Social Sciences, University of Copenhagen, Copenhagen, Denmark; ^4^The National Research Centre for the Working Environment, Copenhagen, Denmark; ^5^Department of Public Health, University of Copenhagen, Copenhagen, Denmark; ^6^Functional Imaging Unit, Department of Clinical Physiology, Nuclear Medicine and PET, Copenhagen University Hospital, Rigshospitalet, Copenhagen, Denmark; ^7^Danish Centre for Sleep Medicine, Department of Clinical Neurophysiology, Copenhagen University Hospital, Rigshospitalet, Copenhagen, Denmark; ^8^Department of Clinical Medicine, Faculty of Health and Medical Sciences, University of Copenhagen, Copenhagen, Denmark; ^9^Mental Health Centre Copenhagen, Copenhagen University Hospital – Mental Health Services CPH, Copenhagen, Denmark

**Keywords:** ultra-high risk of psychosis, white matter, diffusion weighted imaging, sleep, substance use, psychopathology

## Abstract

**Aim:**

White matter changes in individuals at ultra-high risk for psychosis (UHR) may be involved in the transition to psychosis. Sleep-wake disturbances commonly precede the first psychotic episode and predict development of psychosis. We examined associations between white matter microstructure and sleep-wake disturbances in UHR individuals compared to healthy controls (HC), as well as explored the confounding effect of medication, substance use, and level of psychopathology.

**Methods:**

Sixty-four UHR individuals and 35 HC underwent clinical interviews and diffusion weighted imaging. Group differences on global and callosal mean fractional anisotropy (FA) was tested using general linear modeling. Sleep-wake disturbances were evaluated using the subjective measures disturbed sleep index (DSI) and disturbed awakening index (AWI) from the Karolinska Sleep Questionnaire, supported by objective sleep measures from one-night actigraphy. The primary analyses comprised partial correlation analyses between global FA/callosal FA and sleep-wake measures. Secondary analyses investigated multivariate patterns of covariance between measures of sleep-wake disturbances and FA in 48 white matter regions of interest using partial least square correlations.

**Results:**

Ultra-high risk for psychosis individuals displayed lower global FA (*F* = 14.56, *p* < 0.001) and lower callosal FA (*F* = 11.34, *p* = 0.001) compared to HC. Subjective sleep-wake disturbances were significantly higher among the UHR individuals (DSI: *F* = 27.59, *p* < 0.001, AWI: *F* = 36.42, *p* < 0.001). Lower callosal FA was correlated with increased wake after sleep onset (*r* = −0.34, *p* = 0.011) and increased sleep fragmentation index (*r* = −0.31, *p* = 0.019) in UHR individuals. Multivariate analyses identified a pattern of covariance in regional FA which were associated with DSI and AWI in UHR individuals (*p* = 0.028), but not in HC. Substance use, sleep medication and antipsychotic medication did not significantly confound these associations. The association with objective sleep-wake measures was sustained when controlling for level of depressive and UHR symptoms, but symptom level confounded the covariation between FA and subjective sleep-wake measures in the multivariate analyses.

**Conclusion:**

Compromised callosal microstructure in UHR individuals was related to objectively observed disruptions in sleep-wake functioning. Lower FA in ventrally located regions was associated with subjectively measured sleep-wake disturbances and was partly explained by psychopathology. These findings call for further investigation of sleep disturbances as a potential treatment target.

## Introduction

The ultra-high risk state of psychosis (UHR) designates a putative prodromal phase of psychosis (Yung et al., 2005) and is defined by criteria of attenuated psychotic symptoms, brief limited intermittent psychotic symptoms, and/or a diagnosis of schizotypal personality disorder or a genetic risk, along with a functional decline. A recent meta-analysis found a transition rate to psychosis of 25% within the first 3 years (Salazar De Pablo et al., 2021). Multiple risk factors for transitioning has been identified, including severity of attenuated psychotic symptoms, negative symptoms, substance use, physical inactivity, unemployment and male sex (Fusar-Poli et al., 2020). Considering the potential detrimental effects of psychosis, research on modifiable risk factors of disease progression is critical for early intervention (Pantelis et al., 2005; Bora et al., 2009).

The influence of sleep-wake disturbances on the development of psychopathology has increasingly been acknowledged. Sleep-wake disturbances can be evaluated using self-report questionnaires regarding a specific period (e.g., last night or last month), but they are vulnerable to recall-bias (Lunsford-Avery et al., 2015; Aili et al., 2017). Objective methods are polysomnography (the gold standard) and actigraphy, the latter less invasive and more convenient (Aili et al., 2017). Due to a well-described discrepancy between subjective and objective sleep measures, a combination of approaches is preferred (Trimmel et al., 2021). Sleep-wake disturbances are common in individuals diagnosed with schizophrenia (Waite et al., 2020) and commonly precede the first psychotic episode (Yung and Nelson, 2011; Zaks et al., 2022), and have predicted psychotic symptoms in a high-risk sample (Lunsford-Avery et al., 2015). According to a recent systematic review and meta-analysis (Clarke et al., 2021), sleep-wake disturbances are more present in UHR individuals compared to HC, and it applies both to self-reported (e.g., sleep quality and fragmented sleep) and objective sleep-wake measures (e.g., sleep latency, daytime napping, and movement during sleep). However, few studies with objective sleep measures such as actigraphy or polysomnography have been reported, and only four studies were identified and included in the meta-analysis (Clarke et al., 2021).

Cerebral white matter (WM) changes have been shown to be implicated in the pathophysiology of psychotic disorders (Vitolo et al., 2017; Zhao et al., 2022) including UHR individuals, although the changes appear more subtle than in patients with an established psychotic disorder (Peters et al., 2010; Pettersson-Yeo et al., 2011; Karlsgodt et al., 2012; Samartzis et al., 2014; Wheeler and Voineskos, 2014; Canu et al., 2015). The most applied white matter index is fractional anisotropy (FA). Lower FA is often interpreted as impaired WM microstructure (Jones et al., 2013) and have been associated with loss of axonal coherence, demyelination, or neurodegeneration (Song et al., 2002; Alexander et al., 2007, 2011; Pasternak et al., 2009). Lower FA has been reported in UHR individuals compared to HC in widespread WM regions (Carletti et al., 2012; Lagopoulos et al., 2013), such as the major association tracts interconnecting frontal regions with temporal and limbic regions (Clemm Von Hohenberg et al., 2014; Rigucci et al., 2016; Vijayakumar et al., 2016), as well as the large projection tracts connecting the cortical and subcortical structures (Katagiri et al., 2015; Saito et al., 2017). Cross-sectional studies have found regional lower FA to be associated with more severe attenuated psychotic symptoms (Nägele et al., 2020). In a previous independent cross-sectional study in UHR individuals we reported WM alterations in UHR individuals which were associated with positive and negative symptoms as well as level of functioning (Krakauer et al., 2017). In a study population partly overlapping with the current sample we found that global FA at baseline predicted transition to psychosis after one year (Kristensen et al., 2021a), and that changes in negative symptoms were linked to white matter changes in superior longitudinal fasciculus (Kristensen et al., 2021b).

Psychosis has been conceptualized as a global brain disorder of dysconnectivity (Friston, 1998). Global WM studies have identified associations between WM microstructure and sleep-wake measures, such as sleep duration in healthy adults (Khalsa et al., 2017; Grumbach et al., 2020) and in adolescents (Telzer et al., 2015), as well as sleep quality in an aging populations (Kocevska et al., 2019), patients with depression (Sexton et al., 2017), and patients with mild traumatic brain injury (Raikes et al., 2018). However, the participants of these studies are heterogeneous including clinical and non-clinical samples, aging or adolescents, as well as neurologically afflicted populations. Hence the results and localization of the associations are mixed (Zitser et al., 2020). To our knowledge, no studies to date have examined associations between WM and sleep-wake disturbances in UHR individuals.

Global approaches may conceal information derived from informed hypotheses on associations between specific symptoms and predefined WM regions of interest (ROIs). The main commissural tract in the brain is corpus callosum (CC), which is critical for the interhemispheric information transfer and coordination of functional activity (Mancuso et al., 2019). CC has together with neuronal populations located to the brainstem been hypothesized to be involved in a complex synchronization of interhemispheric activity, regulating the timing and duration of rapid eye movement (REM) (Nielsen et al., 1992). Indeed, different aspects of sleep quality have been linked to CC, such as sleep oscillations (Piantoni et al., 2013), sleep efficiency, total sleep time (Altendahl et al., 2020), REM as well as non-REM sleep (Avvenuti et al., 2019; Bernardi et al., 2021). One study found that WM metrics of CC mediated associations between poor sleep quality and symptomatology in patients with depression (Li et al., 2020). Hence, investigating associations between predefined ROIs such as CC and predefined measures of sleep, as well as exploring associations to all WM regions may be optimal.

Substance use disorder is significantly more common in UHR individuals (Carney et al., 2017) and patients suffering from schizophrenia (Hunt et al., 2018) than in the general population. Substance use is known to affect both WM microstructure (Murray et al., 2017; Hampton et al., 2019) and sleep (Huhn et al., 2022), as well as the risk of developing psychosis (Murray et al., 2017; Murrie et al., 2020; Hjorthøj et al., 2021). A meta-analysis found lower FA in CC in individuals with a substance use disorder, however, it appeared dependent on the type of substance (Hampton et al., 2019). Studies have demonstrated that substance use worsens both the subjective experience of sleep quality and objective sleep-wake measures, including reduced total sleep time (TST) and increased Wake After Sleep Onset (WASO) (Huhn et al., 2022). It is well-known that substance use can induce transient and dose dependent psychotic symptoms. A recent meta-analysis reported an overall transition rate of 25% from any substance induced psychosis to schizophrenia with the most elevated transition rate of 34% found in patients with a cannabis-induced psychosis (Murrie et al., 2020).

A study from our group showed that antipsychotic medication affected WM microstructure in frontal fasciculi in antipsychotic naïve patients with first episode psychosis (Ebdrup et al., 2015). Additionally, changes in WM microstructure have been reported in patients with affective disorders (Jenkins et al., 2016; Cui et al., 2020). Hence, the potential confounding effects of both antipsychotic medication, sleep medication, as well as depressive symptoms being markedly prevalent in UHR individuals may be important to explore.

In this study, we examine the association between global and regional WM microstructure and sleep-wake disturbances in UHR individuals compared to HC. We hypothesize, that lower mean global FA as well as lower FA in CC are associated with more severe sleep-wake disturbances. Secondly, we investigate potential covariation between FA in all ROIs and sleep-wake disturbances. Finally, we exploratively investigate if these potential associations may be explained by substance use, medication (antipsychotics and sleep medicine), and psychopathology (depressive symptoms and UHR symptoms).

## Materials and methods

Cross-sectional data were derived from The FOCUS-trial (Glenthøj et al., 2015). The main trial design and primary outcomes have been reported elsewhere (Glenthøj et al., 2015, 2020). After initiation, the main trial was extended with several add-on studies at a later timepoint. Hence, sample size was reduced for the current subset of UHR individuals selected from the main trial (Flow chart in Supplementary Figure 1). Participants were recruited from psychiatric inpatient and outpatient facilities in Copenhagen, Denmark, between April 2014 and December 2017. The trial protocol was approved by the Committee on Health Research Ethics of the Capital Region in Denmark (H-6-2013-015). All participants provided written informed consent prior to inclusion.

### Participants

Data for the current study was collected at baseline from help-seeking individuals meeting at least one of the three UHR-criteria according to the Comprehensive Assessment of At-Risk Mental States (CAARMS) (Yung et al., 2005): attenuated psychotic symptoms, and/or brief limited intermittent psychotic symptoms, and/or trait and vulnerability state along with a significant drop or sustained low functioning for the past year. Exclusion criteria were a history of a psychotic episode of more than one week duration; psychiatric symptoms explained by a physical illness with psychotropic effect (e.g., delirium) or acute intoxication from any substance; a diagnosis of a serious developmental disorder (e.g., Asperger’s syndrome or IQ < 70); or current treatment with methylphenidate. HC were recruited by web based advertising or via advertisements at local educational institutions. HC had no current or previous psychiatric diagnosis, substance use or dependency, and no first-degree relatives with a psychotic disorder. In the main trial HC were concurrently included and matched to the UHR individuals on age, sex, ethnicity, and parental socioeconomic status.

### Assessments

#### Clinical assessments

Axis I and selected Axis II diagnoses (schizotypal-, paranoid-, and borderline personality disorder) were examined using the Structured Clinical Interview for DSM-IV (SCID) (First et al., 1997). To examine whether a participant met the UHR criteria, the semi-structured interview Comprehensive Assessment of At-Risk Mental States (CAARMS) (Morrison et al., 2011) was used. CAARMS is rated with an intensity score and a frequency score in four different domains of positive UHR symptoms (unusual thought content, non-bizarre ideas, disorganized speech, or perceptual abnormalities). Based on these scores it is determined if the patient meets UHR-criteria. If the intensity exceeds cut-off for psychosis and does not resolve spontaneously within the duration of one week, the UHR state is exceeded. Furthermore, UHR criteria of trait and vulnerability is met with a schizotypal personality disorder or a psychotic illness in a first degree relative, along with a decline in functioning (≥30% drop for at least a month) or a sustained low functioning (≤50 for at least a year) assessed with the Social and Occupational Functioning Assessment Scale (SOFAS) (Morosini et al., 2000). Here, we used the CAARMS composite score, which is calculated from the four different domains of positive UHR symptoms by multiplication of the intensity score and the frequency score within each domain, and next adding the scores from the 4 domains. Depressive symptomatology was assessed using the Montgomery-Åsberg Depression Rating Scale (MADRS) (Montgomery and Asberg, 1979), which is a 10 items interview based questionnaire related to depressive symptoms. The Alcohol Smoking and Substance Involvement Screening Test (ASSIST) (Ali et al., 2002) was used to assess the occurrence and extent of substance use. ASSIST is a short interview based questionnaire, consisting of eight questions aiming to examine both present (last three month) and lifetime use of different substances (alcohol, tobacco, cannabis, cocaine, amphetamines, inhalants, sedatives, hallucinogens, opiates, and other drugs). For each substance ever used, frequency was rated on a 5-point Likert scale, (0 = never, 1 = once or twice, 2 = monthly, 3 = weekly and 4 = daily or almost daily). Use of antipsychotics and sleep medicine (including benzodiazepines, antihistamines, and melatonin) was self-reported, both prescribed medicine for daily use and for use “as needed” were registered, and if the medicine was ingested during the 24-h of actigraphy.

All clinical assessments were conducted by experienced psychologists and medical doctors with comprehensive training in the assessment instruments. Inter-rater reliability was assessed in the main trial using intra-class correlations for clinical outcomes, revealing excellent inter-rater reliability (Glenthøj et al., 2020).

#### Sleep assessments

The subjectively experienced sleep quality was assessed with a modified version of the Karolinska Sleep Questionnaire (Åkerstedt et al., 2002) both during the past night and during the past four weeks. Seven items about sleep disturbances were covered: (a) difficulties falling asleep, (b) disturbed/restless sleep, (c) repeated awakenings, (d) premature awakenings, (e) difficulties waking up, (f) non-refreshing sleep, and (g) exhaustion at awakening. All scores ranking from 1 to 5 (1 = never, 2 = seldom, 3 = sometimes/several times per month, 4 = mostly/several days per week, 5 = always), higher scores indicating less refreshing sleep. The Disturbed Sleep Index (DSI) was calculated as the sum of the four items, (a), (b), (c), and (d), ranking from 4 to 20. The Disturbed Awakening Index (AWI) was calculated as the sum of the three items (e), (f), and (g), ranking from 3 to 15. Individuals with missing data on single items were excluded from the analyses.

The objective sleep-wake measures were obtained using an actigraph. An actiwatch (ActiGraph wGT3X-BT from ActiGraph FL, USA) was worn on the non-dominant wrist for 24 h. Data were collected with a sampling rate of 30 Hz and 1-min epochs were used to score wake and sleep pattern. Data were analyzed with ActiGraph Sleep Analysis (ActiGraph, FL, USA). The actigraph measures applied in this study were: total sleep time (TST), wake after sleep onset (WASO) (minutes spend awake after sleep had occurred), sleep efficiency (SE) (the ratio between TST and total time in bed), and sleep fragmentation index (SFI) (index of restlessness during the sleep period expressed as a percentage, higher score indicating more disrupted sleep). The actigraph was accompanied by a sleep diary reporting bedtime, awakening time, and medicine intake.

The subjective sleep measures are regarded as primary in the analyses as they cover the last four weeks and must be considered more representative than one-night actigraphy. In our analyses we include both the subjective and the objective sleep-wake measures and consider whether the objective measures support the findings of the subjective measures.

#### Image acquisition and processing

The details of image acquisition and processing have been described elsewhere (Kristensen et al., 2019). Briefly, we acquired the MRI scans on a 3T scanner (Philips Healthcare, Best, Netherlands). Two diffusion-weighted images using single shot spin-echo echoplanar imaging sequence with 30 non-collinear diffusion-weighted (*b* = 1.000 s/mm^2^) directions and one non-diffusion weighted (*b* = 0 s/mm^2^) in opposite phase encoding directions were acquired, enabling correction for susceptibility distortions (Andersson et al., 2003). Tools from the FSL software library v5.0.10 (Jenkinson et al., 2012) and MRtrix3^1^ was used for image processing. DWI data were denoised (Dhollander et al., 2016; Veraart et al., 2016b,a), and images were corrected for B1 field inhomogeneity (Zhang et al., 2001; Smith et al., 2004). Eddy current and susceptibility artifact correction (Andersson and Sotiropoulos, 2016) was performed, and absolute and relative head motion parameters were extracted. Tract-based spatial statistics (TBSS) (Smith et al., 2004, 2006) was used to align FA data into the FMRIN58 template using the non-linear image registration tool (FNIRT) (Andersson et al., 2007a,b). The mean FA image (threshold of 0.2) was thinned to create mean study-specific FA skeleton maps (Smith et al., 2006). Using the John Hopkins University WM tractography atlas labels (Mori and Van Zijl, 2007; Hua et al., 2008), we calculated mean FA for the 48 ROIs for each UHR individual. Mean global FA was calculated as the average of the weighted FA in the 48 ROIs from skeletonized data. FA for CC was calculated as the summed mean FA for the 3 callosal segments (genu + body + splenium)/3 (Supplementary Figure 2 and Supplementary Text 1 for further details).

MRI quality metrics were assessed by visual inspection, and MRI quality metrics from each subject was calculated using a quality assessment method described by Roalf et al. (2016) [Range between “Good” and “Excellent” quality, details are reported elsewhere (Kristensen et al., 2019)].

### Statistical analyses

#### Univariate analyses

All univariate analyses were performed using Statistical Package for the Social Sciences (SPSS) version 25.0, (IBM, Armonk, NY, USA). Descriptive variables were reported as percent, means, and standard deviations. Chi-square tests and general linear modelling (GLM) were used to compare UHR individuals to HC on descriptive variables. Effect of group on mean global FA and callosal FA were tested using GLM with age, sex, relative and absolute motion in scanner as covariates.

Partial correlation analyses were performed in SPSS to test covariation between mean FA in the CC and the subjective and objective measures of sleep-wake disturbances, including age, sex, relative and absolute motion in scanner as covariates. Primary analyses tested the effect of group with ANOVA (UHR individuals vs. HC). All tests were corrected for multiple comparisons using the Benjamin–Hochberg procedure with an FDR of *p* < 0.05.

*Post-hoc*, we tested the potential effect of substance use, medication, and psychopathology by including each substance as well as a composite score for substance use, medication (antipsychotics and sleep medicine yes/no), and measure of psychopathology [depressive (MADRS score) or UHR-symptoms (CAARMS composite score)] as covariates, along with age, sex, relative and absolute motion in scanner in the partial correlation model. *Post-hoc* tests were uncorrected for multiplicity.

#### Multivariate analyses

Group differences on the 48 ROIs were tested with multivariate GLM using the MATLAB software (version 2021a) including age, sex, relative and absolute motion as covariates.

The partial least square-correlation (PLS-C) analysis (McIntosh and Lobaugh, 2004; Kovacevic et al., 2013) was performed using the MATLAB software (version 2021a). We included two subjective and four objective sleep-wake measures and mean FA values of 48 WM-regions, co-varied for age, sex, and relative and absolute motion in scanner. In brief, PLS-C is used to identify latent variables (LVs), which express maximum covariance between patterns of regional FA associated with sleep-wake disturbances. Both the significance level of the omnibus test (Reisfeld and Mayeno, 2013) and of the individual LVs were assessed using permutation testing (100,000 permutations) to obtain a *p*-value based on non-rotated sampling distribution of singular values (Kovacevic et al., 2013). For the omnibus test, the Inertia index that was calculated as the sum of all singular values of all the LVs identified by PLS-C, was used for permutations testing (Abdi and Williams, 2013). LVs with a *p* < 0.05 were considered significant. Only LVs with a cross-block covariance larger than 5% were reported (Grigg and Grady, 2010). The reliability of saliences was assessed using bootstrapping (100,000 bootstraps with procrustean rotation) to obtain 95% confidence intervals. Confidence intervals of the saliences that did not cross zero were considered reliable (Krishnan et al., 2011). Details on PLS-C are provided in Supplementary Text 2.

In planned exploratory analyses, we entered substance use, medication (antipsychotics and sleep medication), and psychopathology [level of depressive symptoms (MADRS score), and level of UHR-symptoms (CAARMS composite score)] as covariates in the multivariate PLS-C. In addition, we performed sensitivity analyses on a reduced sample of antipsychotic free UHR individuals (*n* = 45). Finally, *post-hoc* analyses explored a potential mediation effects of levels of psychopathology on significant associations between regional FA and sleep-wake measures using Process for SPSS by Hayes (2017).

## Results

Characteristics of participants are reported in Table 1. No differences were found between UHR individuals and HC on the sociodemographic variables of age, sex or estimated premorbid IQ (See further details in Table 1). As well, no differences were found when comparing characteristics of UHR individuals in antipsychotic medication with UHR individuals with no antipsychotic medication (see further details in Supplementary Table 1).

**TABLE 1:** Sociodemographic and clinical data at baseline for individuals at ultra-high risk and healthy controls.

Variable Mean (S.D.)/Percent	UHR (N = 64)	Healthy controls (N = 35)	Significance
**Age** Mean (SD)	23.6 (3.9)	23.7 (2.7)	*p* = 0.87
**Sex**			*p* = 0.07, χ^2^ 2.87
Male	57.8%	40.0%	
Female	42.2%	60.0%	
**Premorbid IQ (DART)**	21.2 (7.2)	22.7 (6.9)	*p* = 0.32
**Years of education**	**13.7 (2.4)**	**16.4 (2.0)**	***p*** **< 0.01**
**Parental SES**			***p*** **= 0.03, χ ^2^ 7.39**
Low	7.8%	0.0%	
Medium	45.3%	23.1%	
High	46.9%	76.9%	
**Function (SOFAS)**	**54.1 (9.9)**	**87.7 (6.8)**	***p*** **< 0.01, *F* = 322.1**
**Activity-level^#^**	**14.9 (17.3)**	**42.7 (8.0)**	***p*** **< 0.01, *F* = 71.96**
**White matter**			
Global, mean FA (SD)	0.599 (0.016)	0.612 (0.012)	***p*** **< 0.001, *F* = 14.56**
Corpus Callosum, mean FA (SD)	0.739 (0.021)	0.753 (0.015)	***p*** **= 0.001, *F* = 11.34**
Absolute motion in scanner	1.262 (0.346)	1.227 (0.373)	*p* = 0.646, *F* = 0.21
Relative motion in scanner	0.178 (0.083)	0.159 (0.063)	*p* = 0.250, *F* = 1.34
**Diagnoses**			
Affective disorder	59.4% (38)	–	
Anxiety disorder	57.8% (37)	–	
Personality disorder	32.4% (21)	–	
Other diagnoses	21.9% (14)	–	
Diagnose of lifetime^†^ abuse	3.1% (2)	–	
Diagnose of lifetime^†^ dependency	9.4% (6)	–	
Diagnose of current^†^ abuse	1.6% (1)	–	
Diagnose of current^†^ dependency	0.0% (0)	–	
**Medication**			
Antipsychotic-naïve	59.4% (38)	–	
Current^†^ antipsychotics	29.7% (19)	–	
Current^†^ antidepressants	23.4% (15)	–	
Current^†^ benzodiazepines	1.6% (1)	–	
Current^†^ melatonin	9.4% (6)	–	
**Clinical symptoms**			
CAARMS composite score	49.91 (15.71)	–	
MADRS total	15.86 (7.31)	–	

^#^Activity-level is hours per week spend on work and education. Significant difference between UHR individuals and healthy controls are marked in bold.

CAARMS, comprehensive assessment of at-risk mental state; DART, Danish adult reading list; FA, fractional anisotropy; IQ, intelligence quotient; MADRS, Montgomery-Åsberg Depression Rating Scale, No., number; SD, standard deviation; SES, socio-economic status; SOFAS, social and occupational function assessment scale; TS, trait and state; UHR, ultra-high risk.

### White matter

UHR individuals had significantly lower global FA (*F* = 14.06, *p* < 0.001), and lower FA in CC (*F* = 12.86, *p* < 0.001) when compared to HC (Table 1).

**FIGURE 1:**
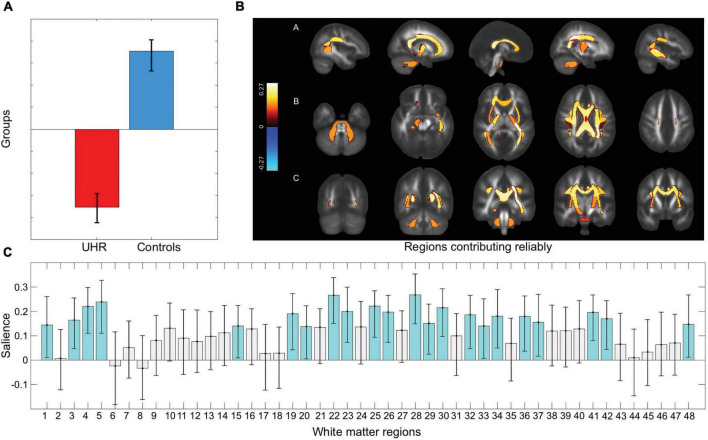
Group difference on 48 white matter regions. Figure displays the results from the multivariate GLM testing group difference between UHR individuals and healthy controls on fractional anisotropy (FA) in 48 regions of interest. **(A)** The significant group difference shows lower fractional anisotropy in UHR individuals compared to healthy controls in the 48 regions of interest (ROIs). **(B)** The ROIs which contributes reliably are visualized using JHU white matter atlas. Reliably contributing regions are bilaterally: middle cerebellar peduncle; genu, body, and splenium of corpus callosum; posterior limb of internal capsule; superior corona radiata; posterior thalamic radiation; external capsule; and superior longitudinal fasciculus. Left hemisphere: retrolenticular part of internal capsule; posterior corona radiata; sagittal stratum; cingulum; and tapetum. Right hemisphere: cerebral peduncle; anterior corona radiata; and cingulum hippocampus. **(C)** The pattern of covariance in all 48 ROIs, with the regions contributing reliably marked in turquoise. A list linking the numbers to the regions are displayed in the Supplementary Text 3. A, anterior; L, left; P, posterior; R, right; UHR, ultra-high risk.

Furthermore, the multivariate test including all 48 ROIs showed significant lower FA in UHR individuals (omnibus test *p* < 0.001). The significant pattern indicated that 22/48 ROIs contributed reliably with lower FA in UHR individuals (*p* = 0.001) bilaterally: middle cerebellar peduncle; genu, body, and splenium of CC; posterior limb of internal capsule; superior corona radiata; posterior thalamic radiation; external capsule; and superior longitudinal fasciculus. Left hemisphere: retrolenticular part of internal capsule; posterior corona radiata; sagittal stratum; cingulum; and tapetum. Right hemisphere: cerebral peduncle; anterior corona radiata; and cingulum hippocampus (Figure 1).

### Sleep

**FIGURE 2:**
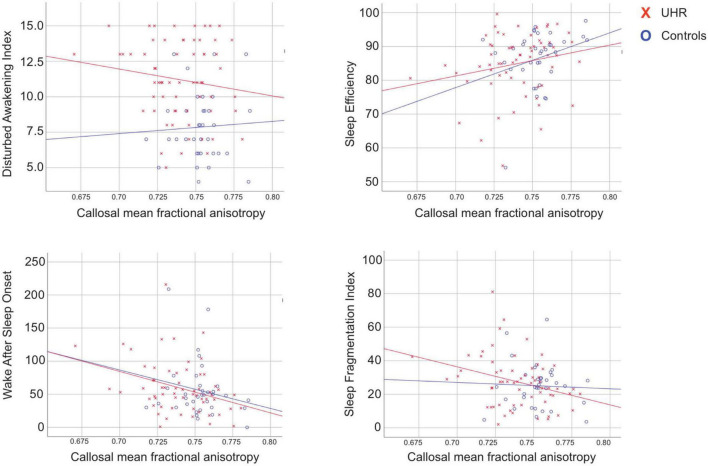
Relations between callosal fractional anisotropy and sleep measures. Scatterplots illustrating the significant relations between mean callosal fractional anisotropy and sleep parameters. UHR, individuals at ultra-high risk for psychosis.

We found a significant difference between groups on the subjective sleep-wake measures (Table 2, Figure 2, and Supplementary Figure 3). The UHR individuals had significantly higher DSI (i.e., more disturbed sleep), both the preceding night (*F* = 18.41, *p* < 0.001) and across the last four weeks (*F* = 27.59, *p* < 0.001). The AWI was significantly higher (i.e., less refreshed at awakening) in the UHR-group, both the preceding night (*F* = 19.03, *p* < 0.001) and across the last four weeks (*F* = 36.42, *p* < 0.001).

**TABLE 2:** Sleep at baseline.

Variable Mean (SD)/percent	UHR individuals (*N* = 64)	Healthy controls (*N* = 35)	Significance
**Karolinska Sleep Questionnaire**			
The last 4 weeks			
Disturbed Sleep Index (DSI)^1^	12.09 (3.56)	8.46 (2.71)	***p*** **< 0.001, F = 27.59**
Disturbed Awakening Index (AWI)^2^	11.20 (2.75)	7.86 (2.42)	***p*** **< 0.001, *F* = 36.42**
The last night			
Disturbed Sleep Index (DSI)^1^	10.66 (3.48)	7.68 (2.83)	***p*** **< 0.001, *F* = 18.41**
Disturbed Awakening Index (AWI)^2^	9.44 (2.51)	7.34 (1.81)	***p*** **< 0.001, *F* = 19.03**
**Actigraphy measures (last night)**			
Total Sleep Time (TST, h/min)^3^	6/39.51 (1/43.01)	6/33.97 (1/5.01)	*p* = 0.775, *F* = 0.08
Sleep Efficiency (SE)^4^	84.90 (9.32)	86.42 (8.42)	*p* = 0.427, *F* = 0.64
Sleep Fragmentation Index (SFI)^5^	27.41 (15.41)	25.07 (13.22)	*p* = 0.453, *F* = 0.57
Wake After Sleep Onset (WASO, min)^6^	59.55 (39.71)	55.74 (42.64)	*p* = 0.662, *F* = 0.19

Table displays the subjective data on disturbed sleep from Karolinska Sleep Questionnaire and objective measures from one-night Actigraphy in ultra-high-risk individuals compared with healthy controls.

^1^Disturbed sleep index (DSI) was computed as the sum of four items [(a)–(d), ranging 1–5, higher scores represented a more disturbed sleep]: (a) difficulties falling asleep, (b) disturbed/restless sleep, (c) repeated awakenings, and (d) premature awakening.

^2^Disturbed awakening index (AWI) was computed as the sum of three items [(e)–(g), all ranging 1–5, higher scores represented poorer sleep (= less refreshing sleep)]: (e) ease of awakening, (f) refreshing sleep, and (g) the degree of exhaustion at awakening.

^3^Total Sleep Time (TST) – The total number of minutes labeled as “asleep” during nighttime using the automatized algorithm from ActiGraph wGT3X-BT.

^4^Sleep Efficiency (SE) – Number of sleep minutes divided by the total number of minutes the subject was in bed.

^5^Sleep Fragmentation Index (SFI) restlessness during the sleep period expressed as sum of Movement Index (MI **=** The total of scored awake minutes divided by Total time in bed in hours × 100) and Fragmentation Index (FI **=** The percentage of one minute periods of sleep vs. all periods of sleep in the sleep period.

^6^Wake after Sleep Onset (WASO): The total number of minutes the subject was awake after sleep onset occurred. Significant difference between UHR individuals and healthy controls are marked in bold.

Overall, we found no significant differences between groups on the objective sleep-wake measures from the actigraph. Among female participants we found significantly higher SE (*F* = 12.83, *p* = 0.001) and TST (*F* = 6.76, *p* = 0.011) and significantly lower WASO (*F* = 8.37, *p* = 0.005) and SFI (*F* = 4.45, *p* = 0.038) (Supplementary Table 2).

### Substance use, psychopathology, and medication

Among the UHR individuals, two had a history and one had a current diagnosis of substance use disorder. Six UHR individuals had an earlier and none had a current diagnosis of dependence, which was in accordance with the eligibility criteria.

UHR individuals had a significantly higher use of nicotine (χ^2^ 18.07, *p* = 0.001) compared to HC, and a significantly lower consumption of alcohol (χ^2^ 9.67, *p* = 0.046). Although not significant, we noticed that three UHR individuals had a daily or almost daily use of cannabis, compared to no HC. Likewise, no HC had ever used simulants (amphetamine, ecstasy, amphetamine, etc.), where three UHR individuals had tried it, and one had a monthly use (Supplementary Table 3).

The UHR individuals comprised a heterogenous sample with a majority of *n* = 38 (59.4%) diagnosed with an affective disorder, *n* = 37 (57.8%) with an anxiety disorder, and *n* = 21 (32.4%) diagnosed with a personality disorder (Table 1). The mean baseline score on MADRS was equivalent to a mild depression, which is in accordance with the high incidence of affective disorders.

Thirty-eight (59.4%) of the sample were antipsychotic-naïve and *n* = 19 (29.7%) reported current use of antipsychotic medication in low dosages. Of other medications which may affect sleep, one UHR individual reported current treatment of a benzodiazepine, 6 melatonin, and 15 reported current use of antidepressants (Table 1).

### Partial correlations between callosal fractional anisotropy and sleep-wake measures

We identified significant negative correlations between callosal FA and WASO (*p* = 0.011, *r* = −0.337) as well as with SFI (*p* = 0.019, *r* = −0.313) in UHR individuals (Table 3). When including all participants, significant correlations between callosal FA and AWI (*p* = 0.029, *r* = −0.230), SE (*p* = 0.020, *r* = 0.243), WASO (*p* = 0.007, *r* = −0.283), and SFI (*p* = 0.032, *r* = −0.225) remained significant after controlling for multiplicity. However, in the within-group analyses we could not confirm the significant correlation between callosal FA, AWI and SE among the UHR individuals, and no significant correlations between global or callosal mean FA were identified in HC. The partial correlation analyses were also performed on the reduced sample of antipsychotic free UHR individuals (*n* = 45), which showed identical results (Supplementary Table 4).

**TABLE 3:** Sleep parameters associated with global and callosal fractional anisotropy.

	Global mean FA			Corpus callosum FA		
		
	UHR	HC	ALL	UHR	HC	ALL
**Karolinska Sleep Questionnaire (the last 4 weeks)**						
**DSI**	*r* = 0.133 *p* = 0.328	*r* = 0.296 *p* = 0.106	*r* = −0.031 *p* = 0.774	*r* = 0.156 *p* = 0.250	*r* = 0.319 *p* = 0.080	*r* = −0.015 *p* = 0.887
**AWI**	*r* = −0.111 *p* = 0.415	*r* = 0.095 *p* = 0.610	***r*** **=** −**0.216** ***p*** **= 0.040**	*r* = −0.173 *p* = 0.203	*r* = 0.231 *p* = 0.211	***r*** **= −0.230** ***p*** = 0.029*
**Actigraphy (last night)**						
**SE**	*r* = 0.166 *p* = 0.221	*r* = 0.212 *p* = 0.251	*r* = 0.186 *p* = 0.078	*r* = 0.225 *p* = 0.096	*r* = 0.303 *p* = 0.097	***r*** **= 0.243** ***p*** = 0.020*
**TST**	*r* = −0.100 *p* = 0.464	*r* = 0.275 *p* = 0.134	*r* = −0.029 *p* = 0.788	*r* = −0.113 *p* = 0.407	*r* = 0.253 *p* = 0.169	*r* = −0.041 *p* = 0.702
**WASO**	***r*** **= −0.295** ***p*** **= 0.027**	*r* = 0.016 *p* = 0.932	***r*** **= −0.215** ***p*** **= 0.041**	***r*** **= −0.337** ***p*** = 0.011*	*r* = −0.165 *p* = 0.376	***r*** **= −0.283** ***p*** = 0.007*
**SFI**	***r*** **= −0.274** ***p*** **= 0.041**	*r* = 0.231 *p* = 0.212	*r* = −0.187 *p* = 0.076	***r*** **= −0.313** ***p*** = 0.019*	*r* = 0.173 *p* = 0.352	***r*** **= −0.225** ***p*** = 0.032*

Table displays results from the partial correlation-analyses between mean global and callosal fractional anisotropy (FA) and sleep parameters, covaried for age, sex, and relative and absolute motion in scanner. Pearson’s correlation tests were two-tailed. Bootstrapping (×1,000) was performed to provide confidence intervals.

Significance level under *p* < 0.05 are marked in bold. *Indicates significant correlation *p* < 0.05 after FDR correction for multiple comparisons according to the Benjamin-Hochberg procedure.

AWI, Disturbed awakening index; CI, confidence interval, DSI, Disturbed sleep index; FA, fractional anisotropy, HC, Healthy controls; r, correlation coefficient; SE, sleep efficiency; SFI, sleep fragmentation index; TST, total sleep time; UHR, individuals at ultra-high risk; WASO, wake after sleep onset.

### Multivariate covariance between fractional anisotropy and sleep-wake disturbances

**FIGURE 3:**
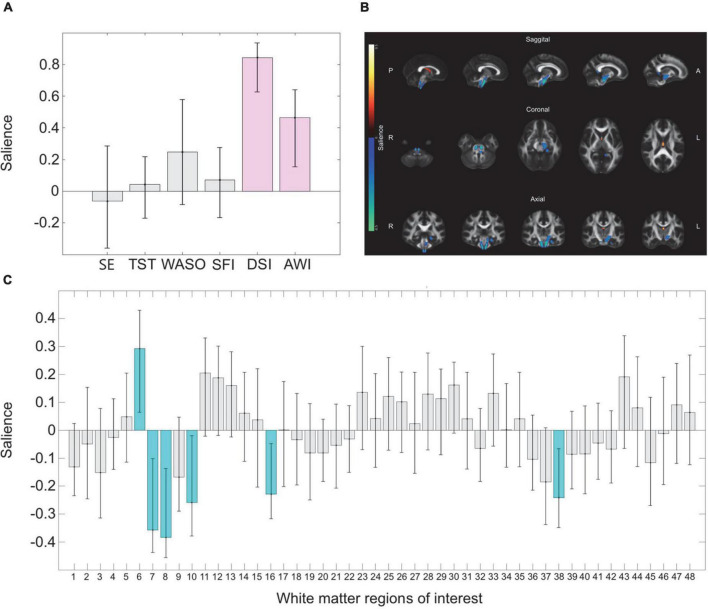
Multivariate correlations between sleep measures and regional fractional anisotropy. Figure displays the results from the multivariate PLS-C analyses on UHR individuals linking covariance in a pattern of sleep disturbances with fractional anisotropy (FA) in 48 regions of interest. **(A)** The pattern of variance six measures of sleep disturbances show that higher disturbed sleep index (DSI) and disturbed awakening index (AWI) marked with purple contributes reliably to the pattern, as the confidence interval does not cross zero. **(B)** The pattern of sleep disturbances is linked to a pattern of regional covariance in FA. The regions of interest which contributes reliably are visualized with the regions contributing reliably projected on a standard brain derived from JHU white matter atlas. Regions colored yellow indicates higher FA, and blue color indicates lower FA. Reliably contributing regions are fornix (higher FA) and left and right corticospinal tract, left cerebral peduncle, left medial lemniscus, and left cingulum (hippocampus) (lower FA). **(C)** The pattern of covariance in all 48 regions of interest, with the regions contributing reliably marked in turquoise. A list linking the numbers to the regions are displayed in the Supplementary Text 3. SE, sleep efficiency; JHU, John Hopkins University; L, left; R, right; SFI, sleep fragmentation index; TST, total sleep time; WASO, wake after sleep onset.

The PLS-C interaction analysis on all participants revealed a trend level significant association between patterns of regional FA and sleep-wake disturbances comparing UHR individuals to HCs (omnibus test *p* = 0.078). *Post-hoc* sensitivity analyses including all HC and a subsample of antipsychotic free UHR individuals showed a strong association (omnibus test *p* = 0.031) between a pattern of disturbed sleep-wake measures (lower SE, higher WASO, SFI, and AWI) and widespread lower FA in 24/48 WM regions explaining 69.89% of the covariance (LV1 *p* = 0.032) (see Supplementary Figure 4A for details). The next step within group PLS-C analysis identified an association between a pattern of regional FA and sleep-wake measures in UHR individuals (omnibus test *p* = 0.028) but not in HC (omnibus test *p* = 0.290). In UHR individuals, one significant latent variable (LV) was identified: LV3 explained 15% of the covariance (*p* = 0.013). LV3 comprised a pattern where higher FA in fornix, along with lower FA in left and right corticospinal tract, left cerebral peduncle, left medial lemniscus, and left cingulum (hippocampus) contributed reliably. This pattern of regional FA was associated with a pattern of more severely disturbed sleep (DSI) and awakening (AWI) contributing reliably (See Figure 3 for details).

### Confounding effects of substance use, medication, and psychopathology

Non-parametric tests of correlations between single substances as well as composite substance score and sleep-wake measures in all participants revealed nicotine use to be positively correlated to scores on 5/6 measures of sleep-wake disturbances: DSI (*p* = 0.003, *r* = 0.292), AWI (*p* < 0.001, *r* = 0.578), SE (*p* = 0.025, *r* = −0.229), WASO (*p* = 0.013, *r* = 0.252), and SFI (*p* = 0.016, *r* = 0.245) (uncorrected). No other substances were correlated to sleep-wake measures (Supplementary Table 5). We did not observe confounding effect of use of single substances or the substance use composite score on the correlation between callosal FA and sleep-wake measures (Supplementary Table 6).

Sleep-wake disturbances were positively associated with psychopathology in UHR individuals: DSI to depressive symptoms (*p* < 0.001, *r* = 0.433) and AWI to UHR-symptoms (*p* = 0.020, *r* = 0.290) (Supplementary Table 7). When *post-hoc* entering psychopathology as well as use of antipsychotics and sleep medication as covariates along with age, sex, relative and absolute motion in scanner in the partial correlation model, we found that the significant negative association between callosal FA and WASO, as well as SFI sustained including antipsychotics (WASO*: p* = 0.011, *r* = −0.337; SFI: *p* = 0.019, *r* = −0.312), sleep medication (WASO*: p* = 0.011, *r* = −0.338; SFI: *p* = 0.020, *r* = −0.310), UHR-symptoms (WASO: *p* = 0.016, *r* = −0.319; SFI: *p* = 0.033, *r* = −0.286), and depressive symptoms (WASO: *p* = 0.010, *r* = −0.339; SFI: *p* = 0.020, *r* = −0.310) as covariates (Supplementary Table 6).

In the multivariate PLS-C, we found no effect of entering substance use, antipsychotic medication, and sleep medication as covariates on the overall result (Supplementary Table 8). However, in the *post-hoc* sensitivity analyses on a reduced sample of antipsychotic free UHR individuals we found a borderline significant association between a pattern of disturbed sleep-wake measures (lower TST, higher DSI) and lower FA in 7/48 WM regions explaining 11.38% of the covariance (omnibus test *p* = 0.033, LV3 *p* = 0.082) (see Supplementary Figure 4B for details). Furthermore, the omnibus test was only significant at trend level when psychopathology was included in the model (UHR-symptoms omnibus test *p* = 0.052. LV3 *p* = 0.005, cross-block covariance 15.97%; Depressive symptoms omnibus test *p* = 0.087, no significant LVs).

## Discussion

To the best of our knowledge, this is the first study in individuals at UHR investigating associations between WM microstructure and subjective and objective sleep-wake measures and how common clinical confounders may affect this association.

As expected, UHR individuals presented with lower FA compared to HC at a global as well as at a regional level in CC. This result was supported in the multivariate test including all 48 ROIs. The finding is in accordance with the majority of studies in UHR individuals reporting subtle and widespread WM alterations (Wheeler and Voineskos, 2014). We identified an increased level of subjective sleep-wake disturbances (DSI and AWI) among UHR individuals compared to HC. These results indicate that both sleep continuity and awakening are disturbed in UHR individuals, congruent with a recent meta-analysis reporting significantly lower subjective sleep quality and significantly more fragmented sleep among high risk individuals (Clarke et al., 2021). We found no group difference regarding the objective measures from the actigraph. Research on objective sleep measures in UHR samples is scarce, as one trial has found significantly reduced SE and significantly higher (i.e., more negatively affected) WASO in UHR individuals compared to HC (Lunsford-Avery et al., 2015), but a recent meta-analysis could not confirm these findings (Clarke et al., 2021) and reported no group differences.

As hypothesized, lower FA in CC was associated with poorer outcomes in objective sleep-wake measures in UHR individuals, although no group difference in the actigraphy outcomes were observed. Lower FA was linked to more time spend awake after sleep had occurred and restlessness during the sleep period, indicating more disrupted sleep in UHR individuals, but not in HC. Nonetheless, when testing all participants, the identical associations remained significant after correction for multiplicity, which may reflect a problem with power due to the smaller sample of HCs. The indication that associations between regional FA and disturbed sleep-wake measures are more pronounced in the patient group than in HC was corroborated by the multivariate PLS-C within-group analyses on all ROIs. Interestingly, this perspective has become increasingly acknowledged, as several studies have concluded that contrary to HC, behavioral measures appear more vigorously associated with and dependent on the structural characteristics of WM in UHR individuals and patients with first-episode schizophrenia (Jessen et al., 2018; Kristensen et al., 2019). A similar finding is reported by Raikes et al. (2018) examining associations between subjectively reported sleep disturbances and FA when comparing patients with mild traumatic brain injury to controls (Raikes et al., 2018). Although they did not identify any group difference on FA, they found that higher regional FA was correlated with better sleep quality in patients with mild traumatic brain injury, but not in the controls.

In the full sample, the subjective sleep-wake measure AWI was significantly correlated with FA in CC but contrary to our expectations we did not find any firm associations between DSI and FA in CC. However, the overall association between sleep-wake disturbances and lower FA in widespread WM regions was corroborated in the sensitivity PLS-C analyses including both HCs and antipsychotic free UHR individuals (Supplementary Figure 4A). Hence, in both HC and UHR individuals in particular SE, WASO, SFI, and AWI appear associated with FA, most pronounced bilaterally and in left hemisphere WM regions located medial and ventrally. When examining UHR individuals and HC separately, the significant association with AWI was lost, likely due to loss of power, as the effect size was similar and the direction of the correlation for the UHR individuals was identical with that of the full sample. In support for this notion, the negative correlation between FA in CC and the objective sleep-wake measure WASO and SFI in the UHR individuals may be considered as corresponding to AWI.

Although it is difficult to compare results across diverse patient populations, Altendahl et al. (2020) have reported callosal FA to be significantly associated with duration of REM-sleep in older adults but could not confirm any links between FA and subjective sleep measures or SE and TST (Altendahl et al., 2020). In another longitudinal study by Telzer et al. (2015) sleep variability in adolescents predicted callosal FA after 1.5 years (Telzer et al., 2015). Both in patients with callosal agenesis (Nielsen et al., 1992) and callosotomy (Avvenuti et al., 2019) studies have indicated that CC plays a vital role for the cross-hemispheric propagation of sleep oscillations and that aberrant callosal FA may potentially interfere with synchronization both in interhemispheric activity as well as in neuronal populations within each hemisphere. Our results confirm the involvement of callosal FA in sleep-wake disturbances as measured by actigraphy in UHR individuals, although we cannot infer any causality due to the cross-sectional design.

The multivariate correlation test revealed a link between the Karolinska sleep measures of DSI and AWI contributing reliably as indicative of sleep-wake disturbances, associated with a pattern of lower FA in 5 ROIs, as well as higher FA in fornix contributing reliably. The ROIs contributing reliably appear mainly to be located ventrally, which is in accordance with previous research locating sleep-wake regulation to sub-thalamic circuits in the brainstem (Tahmasian et al., 2021). We speculate if our findings could be extended to WM connecting deep nuclei structures involved in sleep-wake regulation, such as the brain stem with thalamus, hypothalamus, and basal forebrain. Unfortunately, the current MRI technology does not offer such resolution and our analysis therefore did not include these structures.

Interestingly, FA in CC did not contribute reliably, which indicates that the subjective sleep-wake disturbances may predominantly be linked to other WM networks, compared to the actigraphy measures. Our result may reflect the notion that different aspects of sleep-wake regulation potentially is linked to specialized and differentiated neuronal circuits and corresponding structural networks (Héricé and Sakata, 2019). Sleep-wake regulation involves a complex network including different brain regions and neurotransmitters, and studies have identified that specific damages on different parts of this network result in various types of sleep-wake disturbances (Schwartz and Roth, 2009; Brown et al., 2012; Scammell et al., 2017). Hence, our results may reflect how the discrepancy between self-reported and objective measures of sleep have been interpreted as the measurement of different constructs which present with different biological underpinnings (Rezaie et al., 2018).

The directionality of FA may depend on the patient sample or region of interest (Thomason and Thompson, 2011). FA is an averaged measure derived from the diffusion signal. Hence, being susceptible for extracellular fluids and crossing fibers (Weinberger and Radulescu, 2020), FA lacks microstructural specificity and great caution in the interpretation must be exhibited, as there is no direct correspondence between the MRI-derived measures and the biological underpinnings (Weinberger and Radulescu, 2020). In particular considering the contra intuitive directionality of FA in fornix contributing reliably in the PLS-C, the interpretation must be exhibited cautiously due to fornix’ proximity to cerebrospinal fluid, which could induce volume effects (Kaufmann et al., 2017).

Our results indicate, that although use of nicotine may affect sleep measures, no substance use affected the association between WM and sleep in CC. Previous studies have reported mixed results, a metanalysis found that substance use disorder (alcohol, cocaine and opiates) was associated with FA in CC, but for cannabis and nicotine the results were mixed (Hampton et al., 2019). However, the prevalence of substance use disorder in our sample is limited, and the study was not specifically designed to address this topic, and the result must therefore be interpreted cautiously.

Our exploratory analyses from partial correlations revealed that medication and psychopathology did not confound the association between FA and the measures of WASO and SFI. The result is not surprising, as we did not observe any group difference on these measures comparing UHR individuals to HC. However, the *post hoc* sensitivity PLS-C analyses on antipsychotic-free UHR individuals indicated a potential effect of antipsychotic medication on the association between sleep-wake measures and FA. The pattern of sleep-wake disturbances in antipsychotic free UHR individuals indicated that mainly reduced sleep-time (lower TST) and disturbed sleep (higher DSI) contributed reliably to the covariation with lower regional FA (Supplementary Figure 4B). When UHR individuals on antipsychotic medication were included, sleep time (TST) was no longer contributing reliably to the pattern of sleep-wake disturbances, whereas difficulties in awakening, non-refreshing sleep and exhaustion at awakening (higher AWI) were added as reliable contributors (Figure 3). Although the results with the reduced sample size were only at trend level, we notice that it appeared to reflect the sedative effect antipsychotic medication can have, which lends some external and clinical validity to the finding that antipsychotic medication may confound the association between sleep-wake disturbances and regional FA by inducing more sleep time yet increased sleepiness at awakening. It appears to be recommendable to examine the effect of antipsychotics when investigating sleep disturbances.

Finally, in the multivariate PLS-C, the level of depressive symptoms (MADRS) as well as the level of UHR-symptoms (CAARMS) appeared to confound the association between regional FA and AWI as well as DSI suggesting that the subjective sleep-wake measures may be more susceptible for level of psychopathology. Moreover, the group difference between UHR individuals and HCs on AWI and DSI were highly significant. As a result of this finding, we *post-hoc* explored if level of depressive and UHR-symptoms would mediate the association between regional FA contributing reliably and AWI/DSI (Supplementary Table 9). As one pathway between regional FA and psychopathology were only at trend level (*p* = 0.063), the model did not fulfill requirements for establishing a mediation effect, although all other requirements were met and a significant mediation effect appeared to be present, showing that level of depressive symptoms mediated the association between FA in fornix and DSI. We believe this trend level association may be due to reduced power, hence reflecting a Type 2 error. A recent study demonstrated how diffusion metrics within the CC partially mediated the associations between poor sleep quality/high stress and depressive symptomatology (Li et al., 2020). To confirm the finding of a potential mediating effect of level of psychopathology on the link between WM microstructure and sleep-wake disturbances (which potentially could explain the different associations in patients compared to controls), a study with a larger sample size designed for the purpose needs to be performed.

### Methodological considerations

PLS-C can be difficult to interpret due to the complexity of linking multivariate data patterns. Furthermore, we did not identify any clear interaction effect. However, repeating the within-group analyses from the primary analyses appeared to confirm aspects of our univariate findings and contribute with additional and more specific information on the associations between WM ROIs and sleep-wake disturbances.

A general limitation to the study is the fact that the analyses were secondary to an RCT and this research question was formulated *post-hoc*. Furthermore, the matching between HC and UHR individuals in the main trial was not complete in this subsample. Further, we would optimally have cross-validated our results in an independent sample. As this was not possible, the results must be interpreted cautiously and should be evaluated as hypothesis generating with a need for replication in a larger sample. Nonetheless, we trust the consistent associations across multiple tests to represent a valuable contribution indicative of specific associations between regional WM and sleep in a group of vulnerable patients.

The actigraphy measures were only recorded for a 24-h period, which makes it susceptible to individual and spurious variations. Actigraphy tends to overestimate sleep length and efficiency compared to polysomnography, and overestimation is more pronounced in individuals with sleep-wake disturbances than in healthy individuals (Fekedulegn et al., 2020). To minimize this overestimation, we did supplement the actigraphy with a sleep diary, estimating time in bed more precisely, but unfortunately no data on daytime sleep were obtained. To increase the reliability of actigraphy compared to polysomnography (which determine sleep stages in contrast to actigraphy measures), studies have suggested a minimum of 5–7 consecutive nights of actigraphy, especially when measuring TST (Aili et al., 2017; Fekedulegn et al., 2020). The short duration of the actigraphic measurement reduces its power to detect between-group differences in this study. Furthermore, the one-night actigraphic measurement renders the data sensitive for bias due to lack of habituation to the equipment on the night of examination. This fact might explain why average WASO in the HC in this study was longer [55.74 min (42.64)] than what is usually considered normative in healthy adults (Fekedulegn et al., 2020). This suggests that the primary finding in these UHR individuals are subjectively poor sleep quality. Hence, although the actigraphy data are not optimally valid we nonetheless regard the contribution valuable, as the results appear clinically meaningful as well as mutually confirming.

Strengths of this study comprise the consistent use of relevant covariates along with a meticulous examination of potential confounders. Furthermore, we believe this is the first study to investigate links between WM and sleep-wake disturbances in UHR individuals. Although our findings call for replication in larger samples, our results may contribute with preliminary hypotheses regarding neurobiological underpinnings of a modifiable risk factor for developing psychosis. Future perspectives of examining the relationship between brain structure/functioning and sleep parameters might be suggested to include AI techniques. With this approach multiple sources of information from advanced MR-scans, biological measures of sleep continuity/architecture and subjective measures of sleep quality could be modeled for purposes of prediction and eventually choice of treatment.

## Conclusion

UHR individuals presented with lower global and callosal mean FA compared to HC. In UHR individuals, compromised callosal microstructure was associated with disturbed objectively measured sleep-wake functioning which was not confounded by substance use or medication. Subjectively measured sleep-wake disturbances were associated with a pattern of lower FA in ventrally located WM regions in UHR individuals. This association was not confounded by substance use or medication, but level of depressive and UHR symptoms partly explained it, pointing to a complex interaction between biological factors and psychopathology as determinants of the sleep-wake pattern. These findings suggest sleep disturbances as a potential treatment target, but future longitudinal studies are needed to address the direction of causality.

## Data availability statement

The original contributions presented in this study are included in the article/Supplementary material, further inquiries can be directed to the corresponding author.

## Ethics statement

The studies involving human participants were reviewed and approved by the Committee on Health Research Ethics of the Capital Region in Denmark (H-6-2013-015). The patients/participants provided their written informed consent to participate in this study.

## Author contributions

JRs: analyzing data and writing up results. DN: designing study, preparing dataset, extracting/analyzing actigraphy data, and reviewing/writing. LG: study coordination, collecting data, and reviewing/writing. MJ: processing actigraphy data and reviewing/writing. AG: processing actigraphy data and reviewing/writing. JRg: processing MRI data and reviewing/writing. PJ: reviewing/writing. BG MN, and BE: financing and reviewing/writing. LB: designing study, financing and supervising JRs, and reviewing/writing. TK: conceptualizing and designing study, analyzing data, supervising JRs, and writing up results. All authors contributed to the article and approved the submitted version.

## Footnotes

1
www.mrtrix.org
