# Supplementary material for: White matter microstructure and sleep-wake disturbances in individuals at ultra-high risk of psychosis
Source: Front Hum Neurosci. 2022 Oct 28;16:1029149. doi: 10.3389/fnhum.2022.1029149 (PMC9649829; doi:10.3389/fnhum.2022.1029149)
Supplement: Supplementary file 1 [file Data_Sheet_1.pdf]

Supplementary Material

1 Supplementary Tables

Table S1. Sociodemographic and clinical data at baseline for antipsychotic free individuals at ultra-high risk and UHR individuals on antipsychotic medication

#Activity-level is hours per week spend on work and education. Significant difference between UHR individuals and healthy controls are marked in bold.

Abbreviations: CAARMS: comprehensive assessment of at-risk mental state; DART: Danish adult reading list; FA: fractional anisotropy; IQ: intelligence quotient; MADRS: Montgomery-Åsberg Depression Rating Scale, No.: number; SD: standard deviation; SES: socio-economic status; SOFAS: social and occupational function assessment scale; UHR: ultra-high risk.

**Variable**<br>**Mean (S.D.) / Percent**	**UHR AP FREE**<br>**(N=45)**	**UHR AP**<br>**(N=19)**	**Significance**
**Age** Mean (SD)	23.2 (3.8)	24.5 (4.1)	*p*=0.234
**Sex**			*p*=0.39 χ^2 0.32
Male	67.6 %	32.4 %	
Female	74.2 %	25.9.0 %	
**Premorbid IQ (DART)**	19.8 (6.7)	24.3 (7.7)	***p*=0.023**
**Years of education**	13.6 (2.3)	13.9 (2.8)	*P*=0.629
**Parental SES**			*p*=0.312 χ^2 2.33
Low	11.1%	0.0 %	
Medium	44.4 %	47.4 %	
High	44.4 %	52.6.9 %	
Function (SOFAS)	54.73 (9.9)	52.4 (9.8)	*P*=0.396 F=0.73
Activity-level#	17.1 (17.1)	9.8 (17.1)	*P*=0.128 F=2.38
**White matter**			
Global, mean FA (SD)	0.599 (0.016)	0.602 (0.016)	*p*=0.480 F=0.504
Corpus Callosum, mean FA (SD)	0.739 (0.021)	0.741 (0.021)	*p*=0.731 F=0.73
Absolute motion in scanner	1.313 (0.398)	1.141 (0.103)	*p*=0.070 F=3.41
Relative motion in scanner	0.191 (0.092)	0.147 (0.040)	*p*=0.053 F=3.90

**Diagnoses**			
Affective disorder	66.7% (30)	42.1% (8)	*p*=0.061 χ^2 3.41
Anxiety disorder	57.8% (26)	57.9% (11)	*p*=0.608 χ^2 0.00
Personality disorder	37.8% (17)	21.1% (4)	*p*=0.156 χ^2 1.70
Other diagnoses	24.4% (11)	15.8% (3)	*p*=0.341 χ^2 0.59
Diagnose of lifetime† abuse	4.4% (2)	0.0% (0)	*p*=0.491 χ^2 0.872
Diagnose of lifetime† dependency	6.7 % (3)	15.8% (3)	*p*=0.042 χ^2 1.309
Diagnose of current† abuse	2.2% (1)	0.0% (0)	*p*=0.703 χ^2 0.429
Diagnose of current† dependency	0.0 % (0)	0.0 % (0)	
**Medication**			
Current† antidepressants	24.4% (11)	21.1% (4)	*p*=0.522 χ^2 0.086
Current† benzodiazepines	2.2% (1)	0.0 % (0)	*p*=0.491 χ^2 0.872
Current† melatonin	2.2% (1)	26.3 (5)	***p*=0.007 χ^2 9.128**
**Clinical symptoms**			
CAARMS composite score	47.36 (14.66)	55.94 (14.85)	***p*=0.045 F=4.20**
MADRS total	15.82 (7.70)	15.95 (6.49)	*p*=0.951 F=0.004

Table S2. Effect of sex on sleep parameters

	**Male**<br>**[N] Mean (SD)**	**Female**<br>**[N] Mean (SD)**	**Effect of sex**
**DSI ALL**	[48] 10.71 (3.55)	[50] 10.88 (3.89)	*p*=0.820, F=0.05
**DSI UHR**	[27] 12.15 (3.35)	[36] 12.06 (3.76)	*p*=0.920, F=0.01
**AWI ALL**	[48] 9.67 (2.93)	[51] 10.35 (3.21)	*p*=0.270, F=1.23
**AWI UHR**	[27] 10.78 (2.62)	[37] 11.51 (2.83)	*p*=0.294, F=1.12
**SE ALL**	[46] 82.25 (9.86)	[49] 88.47 (6.92)	***p*=0.001, F=12.83**
**SE UHR**	[25] 80.82 (10.22)	[35] 87.81 (7.49)	***p*=0.003, F=9.37**

**TST ALL**	[46] 373.13 (84.10)	[50] 419.90 (91.51)	***p*=0.011, F=6.76**
**TST UHR**	[25] 364.52 (98.69)	[36] 423.81 (100.16)	***p*=0.026, F=5.231**
**WASO ALL**	[46] 70.13 (49.74)	[49] 46.90 (25.44)	***p*=0.005, F=8.37**
**WASO UHR**	[25] 73.68 (49.86)	[35] 49.46 (27.01)	***p*=0.019, F=5.87**
**SFI ALL**	[46] 29.78 (14.22)	[50] 23.59 (14.50)	***p*=0.038, F=4.45**
**SFI UHR**	[25] 31.19 (14.43)	[36] 24.78 (15.72)	*p*=0.110, F=2.63

Table S3. Substance use in individuals at ultra-high risk for psychosis and healthy controls.

		**UHR-individuals (N), %**	**Healthy controls (N), %**	**Effect size and significance**
**Nicotine**	Never	(29) 45,3%	(29) 82.9%	**χ^2 18.07, *p*=0.001**
	Once or twice	(3) 4.7%	(2) 5.7%	
	Monthly	(1) 1.6%	(1) 2.9%	
	Weekly	(4) 6.3%	(2) 5.7%	
	Daily or almost daily	(27) 42.2%	(1) 2.9%	
**Alcohol**	Never	(10) 15.6%	(2) 5.7%	**χ^2 9.67, *p*=0.046**
	Once or twice	(9) 14.1%	(0) 0.0%	
	Monthly	(23) 34.4%	(19) 54.3%	
	Weekly	(22) 34.4%	(14) 40.0%	

	Daily or almost daily	(1) 1.6%	(0) 0.0%	
**Cannabis**	Never	(43) 67.2%	(24) 68.6%	χ^2 4.06, *p*=0.398
	Once or twice	(9) 14.1%	(7) 20.0%	
	Monthly	(6) 9.4%	(1) 2.9%	
	Weekly	(3) 4.7%	(3) 8.6%	
	Daily or almost daily	(3) 4.7%	(0) 0.0%	
**Stimulants**	Never	(60) 93.8%	(35) 100.0%	χ^2 2.28, *p*=0.320
	Once or twice	(3) 4.7%	(0) 0.0%	
	Monthly	(1) 1.6%	(0) 0.0%	
	Weekly	(0) 0.0%	(0) 0.0%	
	Daily or almost daily	(0) 0.0%	(0) 0.0%	
**Other**	In each category only 1 (Hallucinogens 3) UHR-individuals and 0 healthy controls have used once or twice, and no significant group difference was found.			

Table S4. Post-hoc partial correlation analyses including only antipsychotic-free UHR-individuals

	Global mean FA			Corpus callosum FA		
	**UHR**	**HC**	**ALL**	**UHR**	**HC**	**ALL**
**Karolinska Sleep Questionnaire (the last 4 weeks)**						
**DSI**	r= 0.043<br>*p*= 0.799	r= 0.296<br>*p*= 0.106	r= -0.031<br>*p*= 0.774	r= 0.090<br>*p*= 0.596	r= 0.319<br>*p*= 0.080	r= -0.093<br>*p*= 0.436
**AWI**	r=-0.218<br>*p*= 0.195	r= 0.095<br>*p*= 0.610	**r= -0.216**<br>***p*= 0.040**	r= -0.257<br>*p*= 0.124	r= 0.231<br>*p*= 0.211	**r= -0.273**<br>***p*= 0.020***
**Actigraphy (last night)**						
**SE**	r= 0.291<br>*p*= 0.081	r= 0.212<br>*p*= 0.251	**r= 0.275**<br>***p*= 0.019**	**r= 0.344**<br>***p*= 0.037**	r= 0.303<br>*p*= 0.097	**r= 0.329**<br>***p*= 0.005***
**TST**	r= -0.004<br>*p*= 0.981	r= 0.275<br>*p*= 0.134	r=0.055<br>*p*= 0.644	r= -0.084<br>*p*= 0.621	r= 0.253<br>*p*= 0.169	r= -0.006<br>*p*= 0.963
**WASO**	**r= -0.372**<br>***p*= 0.023**	r= 0.016<br>*p*= 0.932	**r= -0.261**<br>***p*= 0.027**	**r= -0.430**<br>***p*= 0.008***	r= -0.165<br>*p*= 0.376	**r= -0.343**<br>***p*= 0.003***
**SFI**	r= -0.316<br>*p*= 0.057	r= 0.231<br>*p*= 0.212	r= -0.201<br>*p*= 0.091	**r= -0.398**<br>***p*= 0.015***	r= 0.173<br>*p*= 0.352	**r= -0.267**<br>***p*= 0.023***

Table S5. Correlations between substance use and sleep parameters in all participants

	DSI	AWI	SE	TST	WASO	SFI
**Nicotine**	**Rho 0.292** **_p_=0.003**	**Rho 0.578** **_p_<0.001**	**Rho -0.229** **_p_=0.025**	Rho 0.054 *p*=0.603	**Rho 0.252** **_p_=0.013**	**Rho 0.245** **_p_=0.016**
**Alcohol**	Rho -0.071 *p*=0.485	Rho 0.052 *p*=0.608	Rho 0.116 *p*=0.262	Rho 0.157 *p*=0.126	Rho -0.073 *p*=0.477	Rho -0.041 *p*=0.690
**Cannabis**	Rho 0.070 *p*=0.493	Rho 0.026 *p*=0.801	Rho -0.015 *p*=0.882	Rho -0.058 *p*=0.577	Rho -0.047 *p*=0.650	Rho 0.001 *p*=0.998
**Cocaine**	Rho -0.030 *p*=0.772	Rho 0.115 *p*=0.225	Rho -0.072 *p*=0.484	Rho 0.065 *p*=0.528	Rho 0.094 *p*=0.362	Rho 0.076 *p*=0.459
**Stimulants**	Rho 0.197 *p*=0.052	Rho 0.135 *p*=0.183	Rho -0.020 *p*=0.850	Rho -0.033 *p*=0.751	Rho -0.026 *p*=0.802	Rho -0.022 *p*=0.834
**Inhalants**	Rho 0.058 *p*=0.572	Rho 0.133 *p*=0.189	Rho -0.057 *p*=0.579	Rho 0.013 *p*=0.900	Rho -0.057 *p*=0.579	Rho -0.072 *p*=0.485
**Sedatives**	Rho -0.032 *p*=0.751	Rho 0.133 *p*=0.189	Rho -0.135 *p*=0.189	Rho -0.143 *p*=0.166	Rho 0.052 *p*=0.616	Rho 0.120 *p*=0.243
**Hallucinogens**	Rho 0.198 *p*=0.051	Rho 0.109 *p*=0.284	Rho -0.001 *p*=0.993	Rho 0.030 *p*=0.775	Rho -0.069 *p*=0.502	Rho -0.053 *p*=0.606
**Opioids**	Rho -0.032 *p*=0.751	Rho 0.133 *p*=0.89	Rho -0.135 *p*=0.189	Rho -0.143 *p*=0.166	Rho 0.052 *p*=0.616	Rho 0.120 *p*=0.243
**Other**	Rho 0.150 *p*=0.141	Rho 0.096 *p*=0.345	Rho -0.094 *p*=0.360	Rho 0.087 *p*=0.399	Rho 0.143 *p*=0.166	Rho 0.143 *p*=0.166

Table S5 displays the correlations between use of each substance and sleep measures tested nonparametric. Significance level under p<0.05 are marked in bold. P-values are uncorrected for multiple comparisons.

Abbreviations: AWI: Disturbed awakening index; DSI: Disturbed sleep index; Rho: Spearman's correlation coefficient; SE: Sleep efficiency; SFI: Sleep fragmentation index; TST: Total sleep time; WASO: Wake after sleep onset

Table S6. Correlations between sleep parameters and callosal white matter including substance use covariates

ALL participants	CC FA/AWI[a]	CC FA/SE[b]	CC FA/WASO[c]	CC FA/SFI[d]
Model<br>(Significant link between 4 sleep parameters and CC FA)	r= -0.229<br>*p*= 0.025<br>95% CI -0.422—0.044	r= 0.243<br>*p*= 0.020<br>95% CI 0.071-0.403	r= -0.283<br>*p*= 0.007<br>95% CI -0.425—0.107	r= -0.225<br>*p*= 0.032<br>95% CI -0.393—0.020
Nicotine	r= -0.226<br>*p*=0.031<br>95%CI -0.430-0.017	r= 0.236<br>*p*=0.025<br>95%CI 0.067-0,402	r= -0.279<br>*p*=0.007<br>95%CI -0.426—0.119	r= -0.216<br>*p*=0.040<br>95%CI -0.385—0.015
alcohol	r= -0.231<br>*p*=0.028<br>95%CI -0.427-0.016	r= 0.247<br>*p*=0.018<br>95%CI 0.071-0,413	r= -0.289<br>*p*=0.006<br>95%CI -0.460—0.101	r= -0.223<br>*p*=0.034<br>95%CI -0.396—0.035
Cannabis	r= -0.230<br>*p*=0.028<br>95%CI -0.424-0.025	r= 0.244<br>*p*=0.020<br>95%CI 0.079-0,404	r= -0.285<br>*p*=0.006<br>95%CI -0.440—0.121	r= -0.225<br>*p*=0.032<br>95%CI -0.383—0.021
stimulants	r= -0.236<br>*p*=0.024<br>95%CI -0.431-0.028	r= 0.244<br>*p*=0.020<br>95%CI 0.047-0,404	r= -0.282<br>*p*=0.007<br>95%CI -0.422—0.100	r= -0.224<br>*p*=0.033<br>95%CI -0.390—0.025
Inhalants	r= -0.249<br>*p*=0.017<br>95%CI -0.430-0.042	r= 0.241<br>*p*=0.021<br>95%CI 0.071-0,404	r= -0.274<br>*p*=0.009<br>95%CI -0.437—0.119	r= -0.210<br>*p*=0.045<br>95%CI -0.386—0.013
sedatives	r= -0.235<br>*p*=0.025<br>95%CI -0.414-0.039	r= 0.248<br>*p*=0.018<br>95%CI 0.072-0,409	r= -0.283<br>*p*=0.007<br>95%CI -0.423—0.128	r= -0.229<br>*p*=0.029<br>95%CI -0.394—0.044
hallucinogens	r= -0.242<br>*p*=0.021<br>95%CI -0.441-0.043	r= -0.238<br>*p*=0.023<br>95%CI 0.044-0,404	r= -0.275<br>*p*=0.008<br>95%CI -0.424—0.108	r= -0.216<br>*p*=0.039<br>95%CI -0.376—0.007
opioids	r= -0.235	r= -0.248	r= -0.283	r= -0.229

	***p*=0.025**<br>**95%CI -0.420--0.023**	***p*=0.018**<br>**95%CI 0.098-0,417**	***p*=0.007**<br>**95%CI -0.440--0.134**	***p*=0.029**<br>**95%CI -0.395--0.060**
**Substance use composite**	**r= -0.241**<br>***p*=0.019**<br>**95% CI -0.423—0.034**	**r= 0.245**<br>***p*=0.019**<br>**95% CI 0.063-0.415**	**r= -0.288**<br>***p*=0.006**<br>**95% CI -0.450--0.109**	**r= -0.255**<br>***p*=0.032**<br>**95% CI -0.393--0.043**
**UHR individuals**	**CCFA/AWI**	**CCFA/SE**	**CCFA/WASO**	**CCFA/SFI**
**Model**<br>**(Link between 4 sleep parameters and CC FA)**	r= -0.171<br>*p*= 0.203<br>95% CI<br>-0.378-0.078	r= 0.226<br>*p*= 0.091<br>95% CI<br>-0.034-0.425	**r= -0.336**<br>***p*= 0.010**<br>**95% CI**<br>**-0.520--0.127**	**r= -0.310**<br>***p*= 0.019**<br>**95% CI**<br>**-0.507--0.099**
**Antipsychotic medication**	r= -0.170<br>*p*=0.211<br>95%CI<br>-0.404-0.088	r= 0.228<br>*p*=0.091<br>95%CI<br>-0.024-0.450	**r= -0.337**<br>***p*=0.011**<br>**95%CI**<br>**-0.552--0.152**	**r= -0.312**<br>***p*=0.019**<br>**95%CI**<br>**-0.505--0.085**
**Antidepressant medication**	r= -0.155<br>*p*=0.254<br>95%CI<br>-0.369-0.091	r= 0.222<br>*p*=0.100<br>95%CI<br>-0.071-0.439	**r= -0.332**<br>***p*=0.016**<br>**95%CI**<br>**-0.496--0.886**	**r= -0.290**<br>***p*=0.030**<br>**95%CI**<br>**-0.475--0.034**
**Sleep medicine**	r= -0.172<br>*p*=0.204<br>95%CI<br>-0.397-0.069	r= 0.226<br>*p*=0.094<br>95%CI<br>-0.009-0.437	**r= -0.338**<br>***p*=0.011**<br>**95%CI**<br>**-0.527--0.156**	**r= -0.310**<br>***p*=0.020**<br>**95%CI**<br>**-0.520--0.108**
**UHR-symptoms (CAARMS)**	r= -0.123<br>*p*=0.367<br>95%CI<br>-0.353-0.130	r= 0.192<br>*p*=0.156<br>95%CI<br>-0.046-0.419	**r= -0.319**<br>***p*=0.016**<br>**95%CI**<br>**-0.509--0.112**	**r= -0.286**<br>***p*=0.033**<br>**95%CI**<br>**-0.487--0.053**
**Depressive symptoms (MADRS)**	r= -0.176<br>*p*=0.195<br>95%CI<br>-0.388-0.080	r= 0.227<br>*p*=0.092<br>95%CI<br>-0.013-0.440	**r= -0.339**<br>***p*=0.010**<br>**95%CI**<br>**-0.532--0.137**	**r= -0.310**<br>***p*=0.020**<br>**95%CI**<br>**-0.512--0.084**

Table S6 displays results from the test of partial correlation-analyses between mean regional fractional anisotropy (FA) in corpus callosum (CC) and sleep parameters when adding further nuisance variables beyond covarying for age, sex, and relative and absolute motion in scanner. Each correlation tests in all participants added effects of single substance use as well as a composite score for all substance use; in UHR-individuals the effect of medication and psychopathology. Bootstrapping (x1000) was performed to provide confidence intervals.

Significance level under p<0.05 are marked in bold. P-values are uncorrected for multiple comparisons.

Abbreviations: AWI: Disturbed awakening index , CAARMS: comprehensive assessment of at-risk mental state, MADRS: Montgomery-Åsberg Depression Rating Scale, r= correlation coefficient; SE: sleep efficiency, SFI: sleep fragmentation index, UHR: individuals at ultra-high risk for psychosis, WASO: wake after sleep onset

Table S7. Correlations between sleep parameters, clinical symptoms, and medication in UHR

N=63	CAARMS total score	MADRS total score	Antipsychotic treatment	Sleep medication
**DSI**	Rho -0.012 *p*=0.928	**Rho 0.433 *p*<0.001**	Rho 0.099 *p*=0.332	**Rho 0.225 *p*=0.026**
**AWI**	**Rho 0.290 *p*=0.020**	Rho 0.212 *p*=0.092	Rho 0.163 *p*=0.107	**Rho 0.253 *p*=0.012**
**SE**	Rho -0.161 *p*=0.214	Rho -0.070 *p*=0.594	Rho -0.029 *p*=0.781	Rho -0.015 *p*=0.885
**TST**	Rho 0.116 *p*=0.372	Rho 0.102 *p*=0.435	Rho -0.044 *p*=0.671	**Rho 0.206 *p*=0.044**
**WASO**	Rho 0.143 *p*=0.270	Rho 0.096 *p*=0.460	Rho -0.041 *p*=0.695	Rho 0.118 *p*=0.251
**SFI**	Rho 0.111 *p*=0.394	Rho -0.060 *p*=0.643	Rho 0.024 p=0.816	Rho 0.006 *p*=0.951

Table S7 displays the results from nonparametric correlation tests between sleep measures, clinical symptoms and medication in UHR-individuals tested nonparametric with Spearman's Rho. Significance level under p<0.05 are marked in bold. P-values are uncorrected for multiple comparisons.

Abbreviations: AWI: Disturbed awakening index , CAARMS: comprehensive assessment of at-risk mental state, DSI: Disturbed sleep index , MADRS: Montgomery-Åsberg Depression Rating Scale, SE: sleep efficiency, SFI: sleep fragmentation index, TST: total sleep time, UHR: individuals at ultra-high risk for psychosis, WASO: wake after sleep onset

Table S8. Including covariates in the PLS-C

Covariate	Omnibus test	Latent variable	Crossblock covariance
**Substance use composite score**	*p*=0.037	LV3 *p*=0.003	15.65%
**Antipsychotic medication**	*p*=0.025	LV3 *p*=0.018	14.11%
**Sleep medication**	*p*=0.032	LV3 *p*=0.013	14.84%
**Substance use, antipsychotic, and sleep medication**	*p*=0.043	LV3 *p*=0.014	14.47%
**CAARMS score (UHR symptoms)**	*p*=0.052	LV3 *p*=0.005	15.97%.
**MADRS score (depression symptoms)**	*p*=0.087	No significant LVs	n/a
**Substance use, antipsychotic medication, sleep medication, CAARMS, and MADRS scores**	*p*=0.183	No significant LVs	n/a

Table S9. Mediation analyses

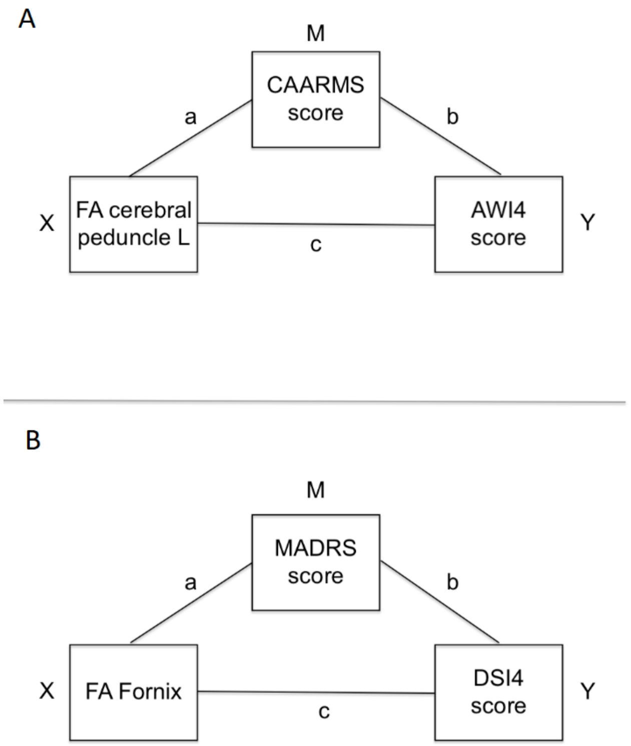

**X**	**Y**	**M**	***a.* estimate** p, effect, (95% CI)	***b.* estimate** p, effect, (95% CI)	***c.* estimate** p, effect, (95% CI)	***c'.* estimate** p, effect, (95% CI)	**Indirect effect** (*a***b*) estimate effect, (95% CI)
**Model A** FA left cerebral peduncle	AWI	CAARMS	***p*=0.048,** **β= -154.817** **(-282.573—27.060)**	*p*=0.059, β= 0.0432 (-0.002-0.088)	***p*=0.037,** **β= -24.227** **(-46.523—1.930)**	*p*=0.130, β= -17.539 (-40.418-5.340)	β= -6.688 95% CI (-16.358-1.536)
**Model B** FA Fornix	DSI	MADRS	*p*=0.063, β= 27.286 95% CI (-1.549-56.121)	***p*<0.001,** **β= 0.198** **95% CI** **(0.088-0.309)**	***p*=0.004,** **β= 19.870** **95% CI** **(6.557-33.183)**	***p*=0.024,** **β= 14.461** **95% CI** **(1.958-26.963)**	**β= 5.409** **95% CI** **(0.062-11.368)**

Table S8 displays the results from the mediation analyses Using Process for SPSS by Hayes. Covariates were age, sex, relative and absolute motion in scanner. 5.000 bootstrap samples were run to obtain 95% confidence interval (CI)

In bold: prerequisites to test for mediation are fulfilled (a, b, or c); and the 95% CI of the indirect effect does not include zero and is therefore significant.

Abbreviations: AWI: Disturbed awakening index , CAARMS: comprehensive assessment of at-risk mental state, DSI: disturbed sleep index, FA: fractional anisotropy, MADRS: Montgomery-Åsberg Depression Rating Scale, UHR: individuals at ultra-high risk for psychosis

2 Supplementary Figures

Figure S1. Flow-chart

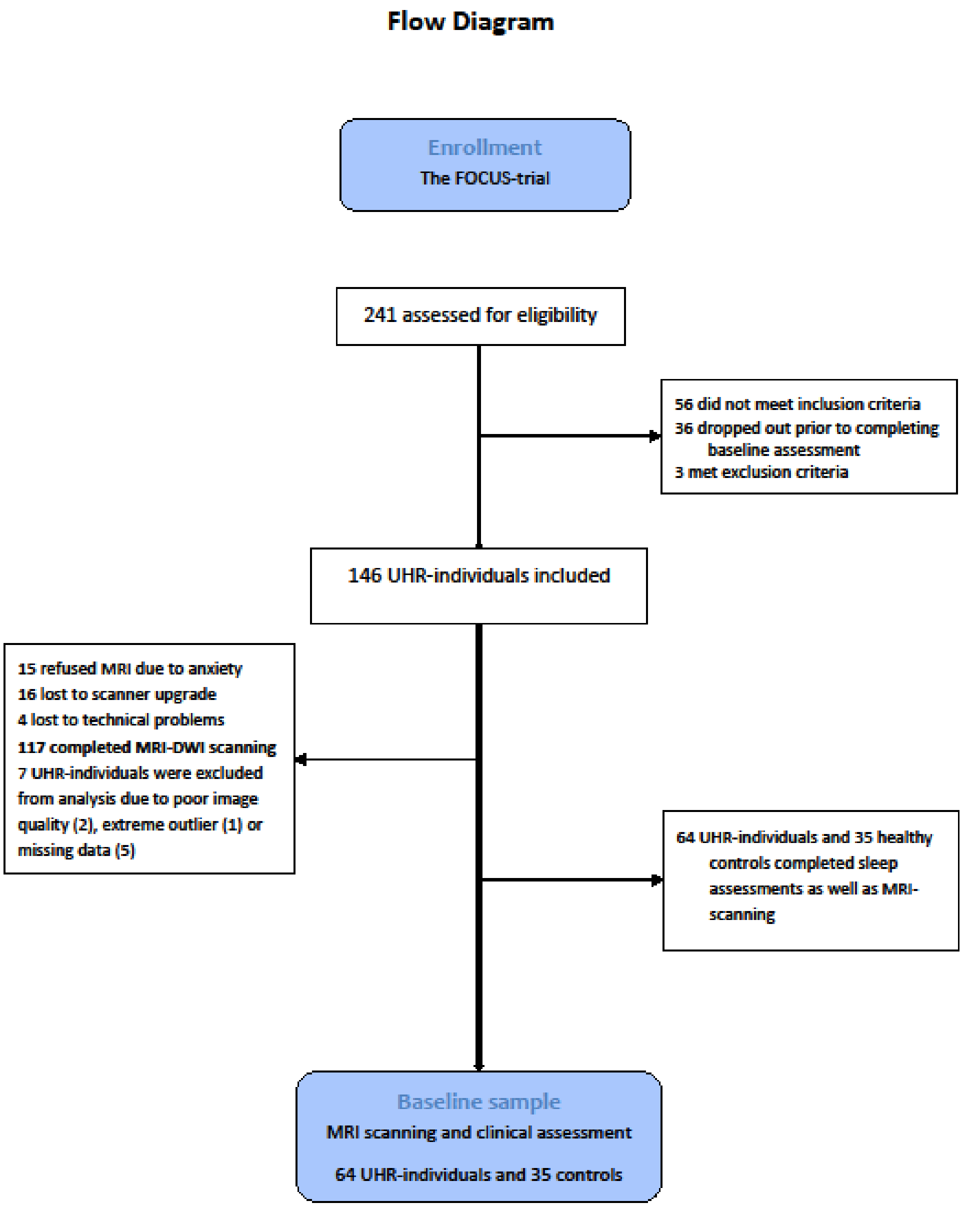

Figure S2. White matter skeleton

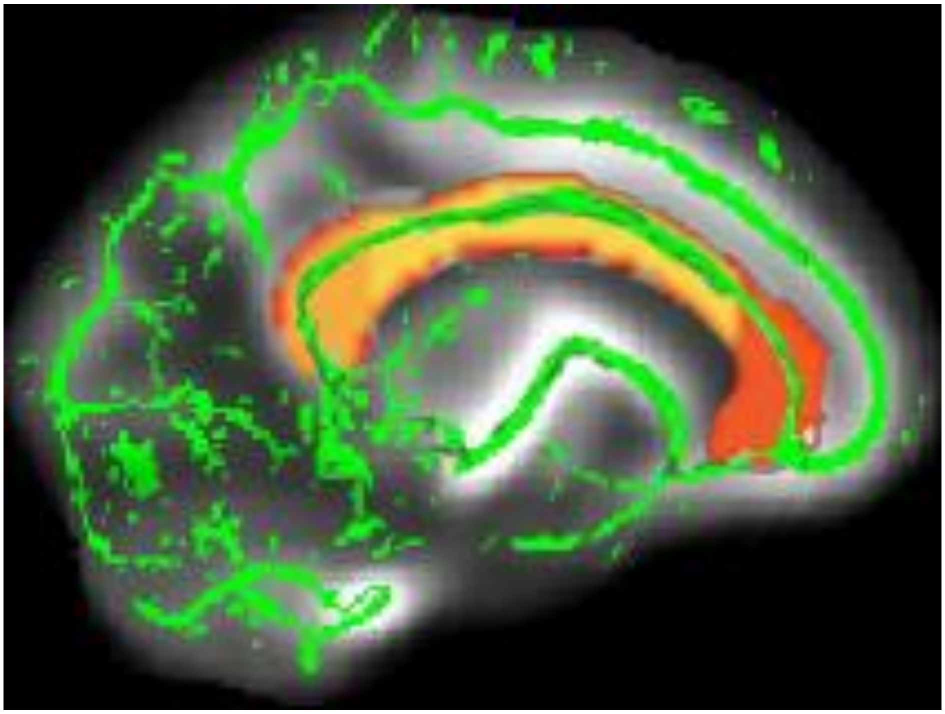

Figure S2. illustrates a) in green the overlaid voxel-based mean FA-skeleton, aligned from participants FA-data and on a standard FA brain template. Tract-based spatial statistics (TBSS) was used to create the study specific mean fractional anisotropy skeleton maps. The FA-skeleton is thinned, representing only the centers of FA-tracts. FA values of all subjects were projected onto the study-specific FA skeleton template. John Hopkins University (JHU) DTI-based white-matter atlas labels was used for extracting mean FA in 48 regions of interest (ROIs) from skeletonized data. B) The location of ROI corpus callosum is shown in red-yellow colors.

Figure S3. Sleep disturbances in UHR-individuals compared to controls

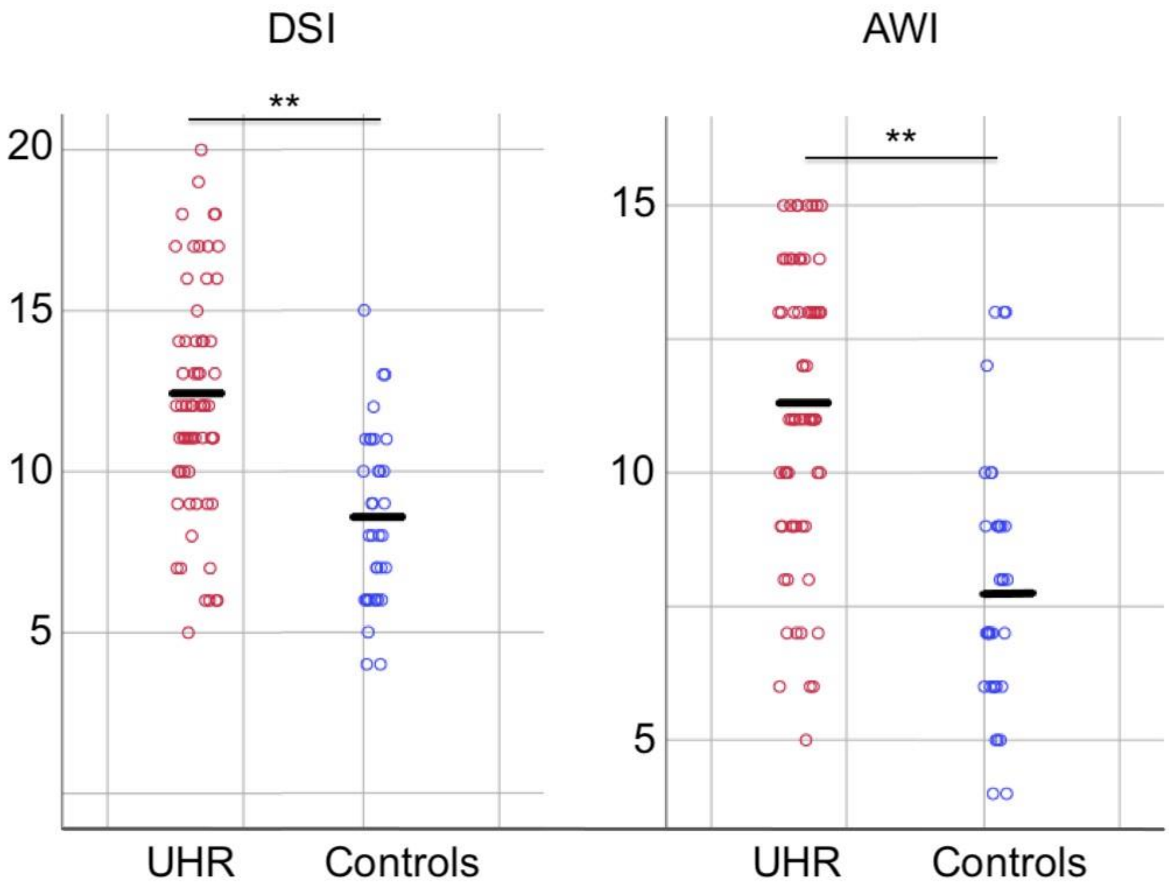

Figure S3. scatterplots demonstrating the group difference on scores of DSI and AWI when comparing UHR-individuals to healthy controls. The mean is indicated by the thick black line. ** indicates significant effect of group below p<0.001.

Abbreviations: AWI: disturbed awakening index, DSI: disturbed sleep index, UHR: individuals at ultra-high risk for psychosis

Figure S4a

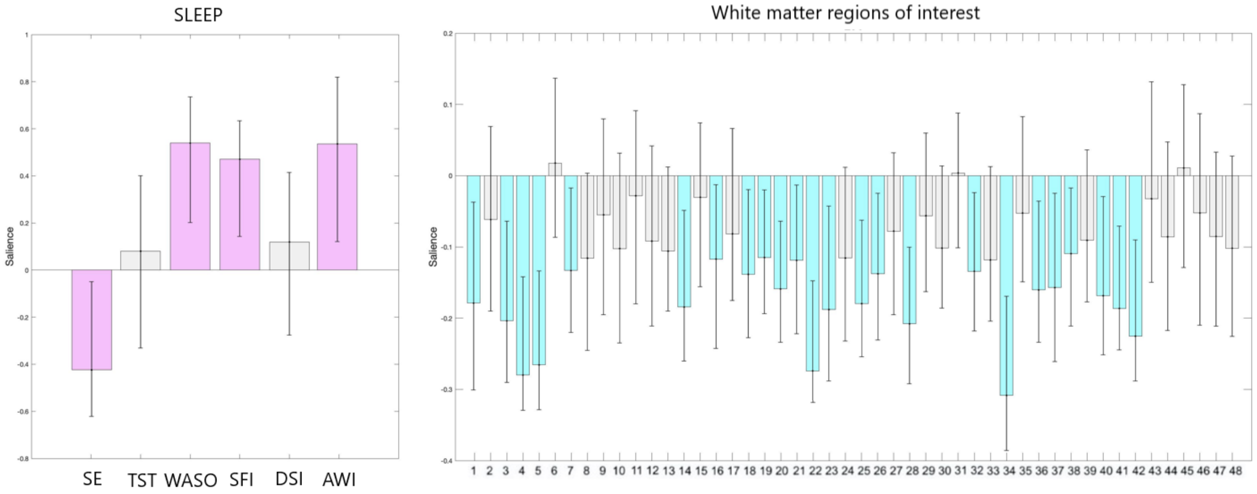

In Figure S4a, all healthy controls (HC, n=35) and antipsychotic free individuals at ultra-high risk for psychosis (UHR n=45) were included in the PLS-C. Omnibus test p=0.031, with one significant LV1 (p=0.032) explaining 69.89% of the covariance between sleep-wake measures and regional FA. At the left, sleep-wake measures contributing reliably to the pattern are colored pink (SE: sleep efficiency, WASO: wake after sleep onset, SFI: sleep fragmentation index, and AWI: disturbed awakening index). On the right side the WM regions contributing reliably are colored turquoise. The pattern displays lower FA in bilaterally: middle cerebellar peduncle; genu-, body-, and splenium of corpus callosum; posterior limb of internal capsule; retrolenticular limb of external capsule; superior corona radiata, cingulum hippocampus, superior longitudinal fasciculus. Left hemisphere: superior cerebellar peduncle, cerebral peduncle, anterior limb of external capsule, posterior corona radiata, saggital stratum, external capsule, cingulum cingulate, fornix cres stria terminalis. Right hemisphere: corticispinal tract, and anterior corona radiata.

Figure S4b

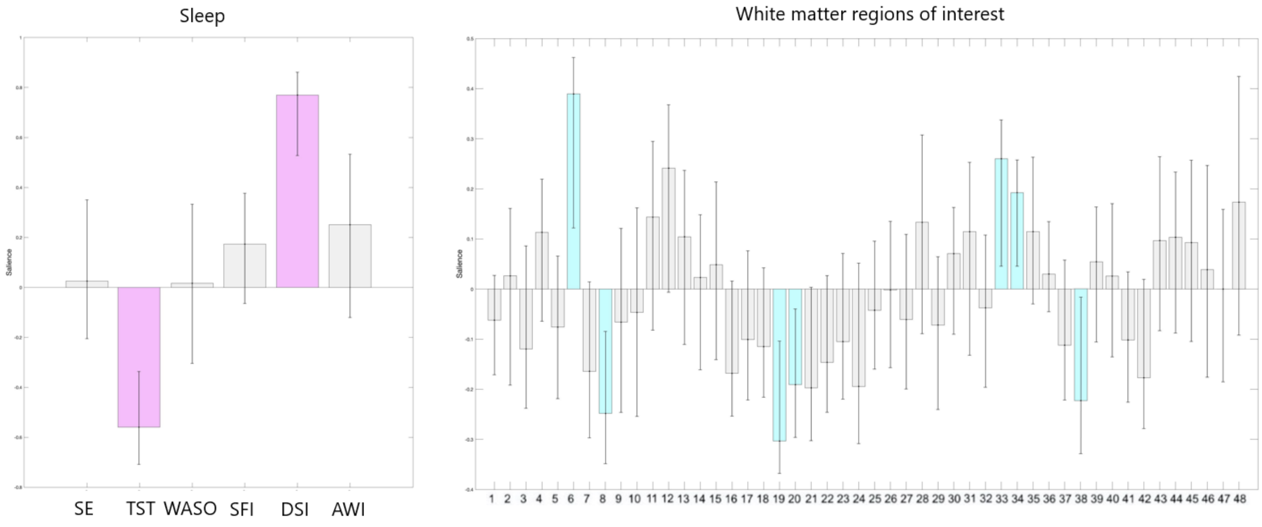

In Figure S4b, antipsychotic free individuals at ultra-high risk for psychosis (UHR n=45) were included in the PLS-C. Omnibus test *p*=0.033, with no significant LVs. Displayed is the borderline significant LV3 (*p*=0.082) explaining 11.38% of the covariance between sleep-wake measures and regional FA. At the left, sleep-wake measures contributing reliably to the pattern are colored pink (TST: total sleep time, and DSI: disturbed sleep index). On the right side the WM regions contributing reliably are colored turquoise. The pattern displays higher FA in fornix and left and right external capsule, and lower FA in left corticospinal tract, left and right posterior limb of internal capsule, and left cingulum hippocampus.

3 Supplementary Text

Text S1. Image acquisition and processing

Magnetic resonance imaging (MRI) scans were acquired on a 3 tesla scanner (Philips Healthcare, Best, The Netherlands), using a 32-channel SENSE head coil (In vivo, Orlando, Florida, USA). Diffusion-weighted images (DWI) were acquired using single shot spin-echo echoplanar imaging (EPI) sequence with 30 non-collinear diffusion weighted (b = 1000 s/mm^2) scans and 5 diffusion unweighted (b = 0 s/mm^2) scans. Other acquisition settings were: Acquisition matrix = 128 × 99 × 75; voxel dimensions = 1.88 × 2.41 × 2mm^3, no slice gap=0; reconstructed voxel dimensions = 1.88 x 1.88 x 2mm$^{3;}$ TR=7058 ms; TE = 68 ms; parallel imaging SENSE factor = 3(AP); flip angle = 90°. Two DWI scans were acquired, where an opposite phase encoding direction were used in the second scan, enabling correction for susceptibility distortions[1,2]. Tools from the FSL software library v5.0.10[3] and MRtrix3 (www.mrtrix.org) were used for image processing. Non-brain tissue was removed using *dwi2mask*[4]. DWI data were denoised by exploiting data-redundancy in the principal component analyses (PCA) domain[5,6]. Next, we performed B1 field inhomogeneity correction[7,8]. An eddy current and susceptibility artifact correction was performed using *topup*[9] and *eddy*[9]. We extracted absolute and relative head motion parameters from *eddy_restricted_movement_rms* output of *eddy*. We used the motion parameters to correct for head motion in the statistical analyses. Diffusion (kurtosis) tensor were computed using rotated diffusion vectors and iterative reweighted linear least squares estimator[10]. Diffusion parameter maps of fractional anisotropy (FA) were calculated using *tensor2metric*[11]. Tract-based spatial statistics (TBSS)[7,12] was used to align FA data using the nonlinear image registration tool (FNIRT)[13,14]. The resulting mean FA image (threshold of 0.2) was thinned to create a mean study-specific FA skeleton template. Next, the nearest maximum FA values of all subjects were projected onto the mean study-specific FA skeleton template[12]. Using the John Hopkins University (JHU) DTI-based white-matter (WM) atlas labels[15,16], we extracted the mean FA values in 48 WM label regions of interest (ROIs) from skeletonized data. Quality control was done by visually inspecting all DW images slice by slice before processing. Prior to statistical analyses, FA images were visually inspected, and excluded if the image quality was judged to be of poor quality. Three image quality metrics (temporal signal-to-noise ratio (TSNR), maximum voxel intensity outlier count (MAXVOX) and mean voxel intensity outlier count (MEANVOX)) were calculated from each subjects DW image using a quality assessment method described in Roalf et al[17]. The measured quality metrics in this study ranged between the 'good' and 'excellent' quality.

Supplementary Text S2. Partial Least Square Correlation analysis

Partial least square correlation (PLS-C) is a multivariate analysis method[18] with advantageous features in modeling complex associations, such as interactions and correlations between multiple behavioral and neuroimaging data[19]. Moreover, as the effect sizes of the WM-alterations in UHR-individuals are expected to be small, univariate statistical analysis are vulnerable to type-2 errors, when correction for multiple comparisons is applied. Applying PLS-C, we expect a larger sensitivity to obtain additional and complementary information on the complex relations between the subtle WM changes and sleep disturbances in UHR-individuals. We have applied similar PLS-C approaches in independent studies of UHR-individuals[20,21] and first-episode psychotic patients[22,23].

In the current study, we included 6 measures of sleep quality and mean fractional anisotropy (FA) values of 48 white matter (WM) regions. First, a correlation matrix was computed between sleep measures and FA data. These correlation matrixes were calculated for each group (UHR and HC). Secondly, the correlation matrix was decomposed using singular value decomposition (SVD) to obtain latent variables (LVs). The latent variables express the maximum covariance between patterns of sleep measures and group-specific regional FA. Significance of the latent variables was determined through permutation testing. The numerical order (LV1, LV2…) of the LVs is based on the portion of the covariance matrix they explain. All LVs combined completely describe 100% of the covariance matrix. If the correlation is evident enough, then the LV is significant (capturing the part of the covariance, where the regional FA is associated to sleep measures). Thus, the size of the LV and its significance are two unrelated measures. By projecting a significant latent variable back to the original variables, the contribution of each of the sleep measures and ROIs to the latent variable can be determined. These projections are known as saliences. Bootstrapping was used to determine if the contribution of a sleep measure or ROI was reliable.

For visual purpose, we projected regions where reliable contributions on FA was found, on to a standard FA brain template from the FSL-JHU-DTI-based white-matter atlas[16].

Supplementary text S3. Labels of the 48 white matter regions from the FSL-JHU-DTI-based white-matter atlas

1. Middle cerebellar peduncle
2. Pontine crossing tract
3. Genu of corpus callosum
4. Body of corpus callosum
5. Splenium of corpus callosum
6. Fornix
7. Corticospinal tract R
8. Corticospinal tract L
9. Medial lemniscus R
10. Medial lemniscus L
11. Inferior cerebellar peduncle R
12. Inferior cerebellar peduncle L
13. Superior cerebellar peduncle R
14. Superior cerebellar peduncle L
15. Cerebral peduncle R
16. Cerebral peduncle L
17. Anterior limb of internal capsule R
18. Anterior limb of internal capsule L
19. Posterior limb of internal capsule R
20. Posterior limb of internal capsule L
21. Retrolenticular part of internal capsule R
22. Retrolenticular part of internal capsule L
23. Anterior corona radiata R
24. Anterior corona radiata L
25. Superior corona radiata R
26. Superior corona radiata L
27. Posterior corona radiata R
28. Posterior corona radiata L
29. Posterior thalamic radiation R
30. Posterior thalamic radiation L
31. Sagittal stratum R
32. Sagittal stratum L
33. External capsule R
34. External capsule L
35. Cingulum cingulate gyrus R
36. Cingulum cingulate gyrus L
37. Cingulum hippocampus R
38. Cingulum hippocampus L
39. Fornix cres Stria terminalis R
40. Fornix cres Stria terminalis L
41. Superior longitudinal fasciculus R
42. Superior longitudinal fasciculus L
43. Superior frontooccipital fasciculus R
44. Superior frontooccipital fasciculus L
45. Uncinate fasciculus R
46. Uncinate fasciculus L
47. Tapetum R
48. Tapetum L

1. Skare S, Andersson JL. On the effects of gating in diffusion imaging of the brain using single shot EPI. *Magn Reson Imaging*. 2001;19(8):1125-1128. doi:10.1016/S0730-725X(01)00415-5

2. Andersson JLR, Skare S, Ashburner J. How to correct susceptibility distortions in spin-echo echo-planar images: Application to diffusion tensor imaging. *Neuroimage*. 2003;20(2):870-888. doi:10.1016/S1053-8119(03)00336-7

3. Jenkinson M, Beckmann CF, Behrens TEJ, Woolrich MW, Smith SM. Fsl. *Neuroimage*. 2012;62(2):782-790. doi:10.1016/j.neuroimage.2011.09.015

4. Dhollander T, Raffelt D, Connelly A. Unsupervised 3-tissue response function estimation from single-shell or multi-shell diffusion MR data without a co-registered T1 image. *ISMRM Work Break Barriers Diffus MRI*. 2016;35(September):5. https://www.researchgate.net/publication/307863133_Unsupervised_3-tissue_response_function_estimation_from_single-shell_or_multi-shell_diffusion_MR_data_without_a_co-registered_T1_image

5. Veraart J, Novikov DS, Christiaens D, Ades-aron B, Sijbers J, Fieremans E. Denoising of diffusion MRI using random matrix theory. *Neuroimage*. 2016;142(November 15):394-406. doi:10.1016/j.neuroimage.2016.08.016.

6. Veraart J, Fieremans E, Novikov DS. Diffusion MRI noise mapping using random matrix theory. *Magn Reson Med*. 2016;76(5):1-12. doi:10.1002/mrm.26059.

7. Smith SM, Jenkinson M, Woolrich MW, et al. Advances in functional and structural MR image analysis and implementation as FSL. *Neuroimage*. 2004;23(S1)(SUPPL. 1):208-219. doi:10.1016/j.neuroimage.2004.07.051

8. Zhang Y, Brady M, Smith S. Segmentation of brain MR images through a hidden Markov random field model and the expectation-maximization algorithm. *IEEE TransMedImaging*. 2001;20(0278-0062):45-57. doi:10.1109/42.906424

9. Andersson JLR, Sotiropoulos SN. An integrated approach to correction for off-resonance effects and subject movement in diffusion MR imaging. *Neuroimage*. 2016;125:1063-1078. doi:10.1016/j.neuroimage.2015.10.019

10. Veraart J, Sijbers J, Sunaert S, Leemans A, Jeurissen B. Weighted linear least squares estimation of diffusion MRI parameters: Strengths, limitations, and pitfalls. *Neuroimage*. 2013;81:335-346. doi:10.1016/j.neuroimage.2013.05.028

11. Basser PJ, Mattiello J, LeBihan D. MR diffusion tensor spectroscopy and imaging. *Biophys J*. 1994;66(1):259-267. doi:10.1016/S0006-3495(94)80775-1

12. Smith SM, Jenkinson M, Johansen-Berg H, et al. Tract-based spatial statistics: Voxelwise analysis of multi-subject diffusion data. *Neuroimage*. 2006;31(4):1487-1505. doi:10.1016/j.neuroimage.2006.02.024

13. Andersson JLR, Jenkinson M, Smith S. Non-linear registration, aka spatial normalisation. FMRIB Technial Report TR07JA2. *Oxford Cent Funct Magn Reson Imaging Brain, Dep Clin Neurol Oxford Univ Oxford, UK*. 2007;(June):22. doi:10.1016/j.neuroimage.2008.10.055

14. Andersson JLR, Jenkinson M, Smith SM. Non-linear optimisation. FMRIB technical report TR07JA1. *In Pract*. 2007;(June):16.

http://fsl.fmrib.ox.ac.uk/analysis/techrep/tr07ja1/tr07ja1.pdf

15. Mori S, Zijl P Van. Human white matter atlas. *Am J Psychiatry*. 2007;164(July):75390. doi:10.1176/appi.ajp.164.7.1005

16. Hua K, Zhang J, Wakana S, et al. Tract Probability Maps in Stereotaxic Spaces: Analyses of White Matter Anatomy and Tract-Specific Quantification. *Neuroimage*. 2008;39(1):336-347. doi:10.1055/s-0029-1237430.Imprinting

17. Roalf DR, Quarmley M, Elliott MA, et al. The Impact of Quality Assurance Assessment on Diffusion Tensor Imaging Outcomes in a Large-Scale Population-Based Cohort. *Neuroimage*. 2016;125:903-919. doi:10.1016/j.neuroimage.2015.10.068.

18. Krishnan A, Williams LJ, McIntosh AR, Abdi H. Partial Least Squares (PLS) methods for neuroimaging: A tutorial and review. *Neuroimage*. 2011;56(2):455-475. doi:10.1016/j.neuroimage.2010.07.034

19. Sarstedt M, Hair JF, Ringle CM, Thiele KO, Gudergan SP. Estimation issues with PLS and CBSEM: Where the bias lies! *J Bus Res*. 2016;69(10):3998-4010. doi:10.1016/j.jbusres.2016.06.007

20. Krakauer K, Ebdrup BH, Glenthoj BY, et al. Patterns of white matter microstructure in individuals at ultra-high-risk for psychosis: Associations to level of functioning and clinical symptoms. *Psychol Med*. 2017;47(15):2689-2707. doi:10.1017/S0033291717001210

21. Kristensen TDTD, Mandl RCWRCW, Raghava JMJM, et al. Widespread higher fractional anisotropy associates to better cognitive functions in individuals at ultra-high risk for psychosis. *Hum Brain Mapp*. 2019;40(June):hbm.24765. doi:10.1002/hbm.24765

22. Jessen K, Mandl RCW, Fagerlund B, et al. Patterns of cortical structures and cognition in antipsychotic-naïve patients with first-episode schizophrenia: A partial least squares correlation analysis. *Biol Psychiatry Cogn Neurosci Neuroimaging*. Published online September 25, 2018. doi:10.1016/J.BPSC.2018.09.006

23. Thomas MB, Raghava JM, Pantelis C, et al. Associations between cognition and white matter microstructure in first-episode antipsychotic-naïve patients with schizophrenia and healthy controls: A multivariate pattern analysis. *Cortex*. 2021;139:282-297. doi:10.1016/j.cortex.2021.03.003
